# Realization of real-time X-ray stereoscopic vision during interventional procedures

**DOI:** 10.1038/s41598-018-34153-9

**Published:** 2018-10-26

**Authors:** Kai Deng, Bo Wei, Mo Chen, Zhiyin Huang, Hao Wu

**Affiliations:** 1 Department of Gastroenterology, West China Hospital, Sichuan University, 37 Guoxue Lane, 610041 Chengdu, Sichuan Province China; 2 Department of Gastroenterology, Tibetan Chengdu Branch Hospital of West China Hospital, Sichuan University, No. 20, Heng Street, Ximian Bridge, 610041 Chengdu, Sichuan Province China

## Abstract

During interventional procedures, the deficiencies of nonstereoscopic vision increase the difficulty of identifying the anteroposterior direction and pathways of vessels. Therefore, achieving real-time stereoscopic vision during interventional procedures is meaningful. Pairs of X-ray images were captured with identical parameter settings, except for different rotation angles (represented as the α angle). The resulting images at these α angles were used as left-eye and right-eye views and were horizontally merged into single left-right 3D images. Virtual reality (VR) glasses were used for achieving stereo vision. Pairs of X-ray images from four angiographies with different α angles (1.8–3.4°) were merged into left-right 3D images. Observation with VR glasses can produce realistic stereo views of vascular anatomical structure. The results showed that the optimal α angles accepted by the brain for generating stereo vision were within a narrow range (approximately 1.4–4.1°). Subsequent tests showed that during transcatheter arterial chemoembolization, 3D X-ray stereoscopic images provided significantly improved spatial discrimination and convenience for identifying the supply vessels of a liver tumor and its anteroposterior direction compared with plain X-ray images (all *P* < 0.01). Real-time X-ray stereoscopic vision can be easily achieved via the straightforward method described herein and has the potential to benefit patients during interventional procedures.

## Introduction

Interventional procedures are typically performed using X-rays, computed tomography (CT), ultrasound, or other imaging modalities^1^. When using X-ray fluoroscopy^2^ (involving an imaging device employing X-rays to obtain real-time moving images of the patients’ body) (https://en.wikipedia.org/wiki/Fluoroscopy. Accessed July 23, 2018), a planar perspective view is available, but stereoscopic vision is not. Without this spatial sense, the anteroposterior position and direction are difficult to determine, so the angle and orientation must be repeatedly adjusted to facilitate the exploration and validation of appropriate directions and paths during interventional procedures. These visualization challenges increase the operative time and the cumulative dose of radiation. The spatial sense is essential for identifying the spatial structure of sectional anatomy to discriminate among overlapping images and determine the anteroposterior direction and pathways for particular interventional procedures, such as transjugular intrahepatic portosystemic shunt^2^ (TIPS, an interventional procedure for creating an artificial channel between the hepatic and portal veins) (https://en.wikipedia.org/wiki/Transjugular_intrahepatic_portosystemic_shunt. Accessed July 23, 2018), transcatheter arterial chemoembolization (TACE, an interventional procedure for restricting tumor’s blood supply to induce the ischemic tumor necrosis) (https://en.wikipedia.org/wiki/Transcatheter_arterial_chemoembolization. Accessed July 23, 2018), some interventional cardiac operations (recanalization of chronic total occlusion of the coronary arteries), and some orthopedic operations, etc. Sometimes, the variation in the direction of vascular openings will greatly increase the difficulty of the selective insertion of a guidewire and catheter without stereo vision. Therefore, achieving real-time 3D vision during interventional procedures is necessary and meaningful. Three-dimensional reconstruction, virtual reality (VR), mixed reality (MR) and augmented reality (AR) technology have given rise to significant concern in many fields, such as education, medicine, movies, games and industrial design^3–9^. This stereo-imaging technology is beginning to be introduced into the medical field^10^.

**Figure 1:**
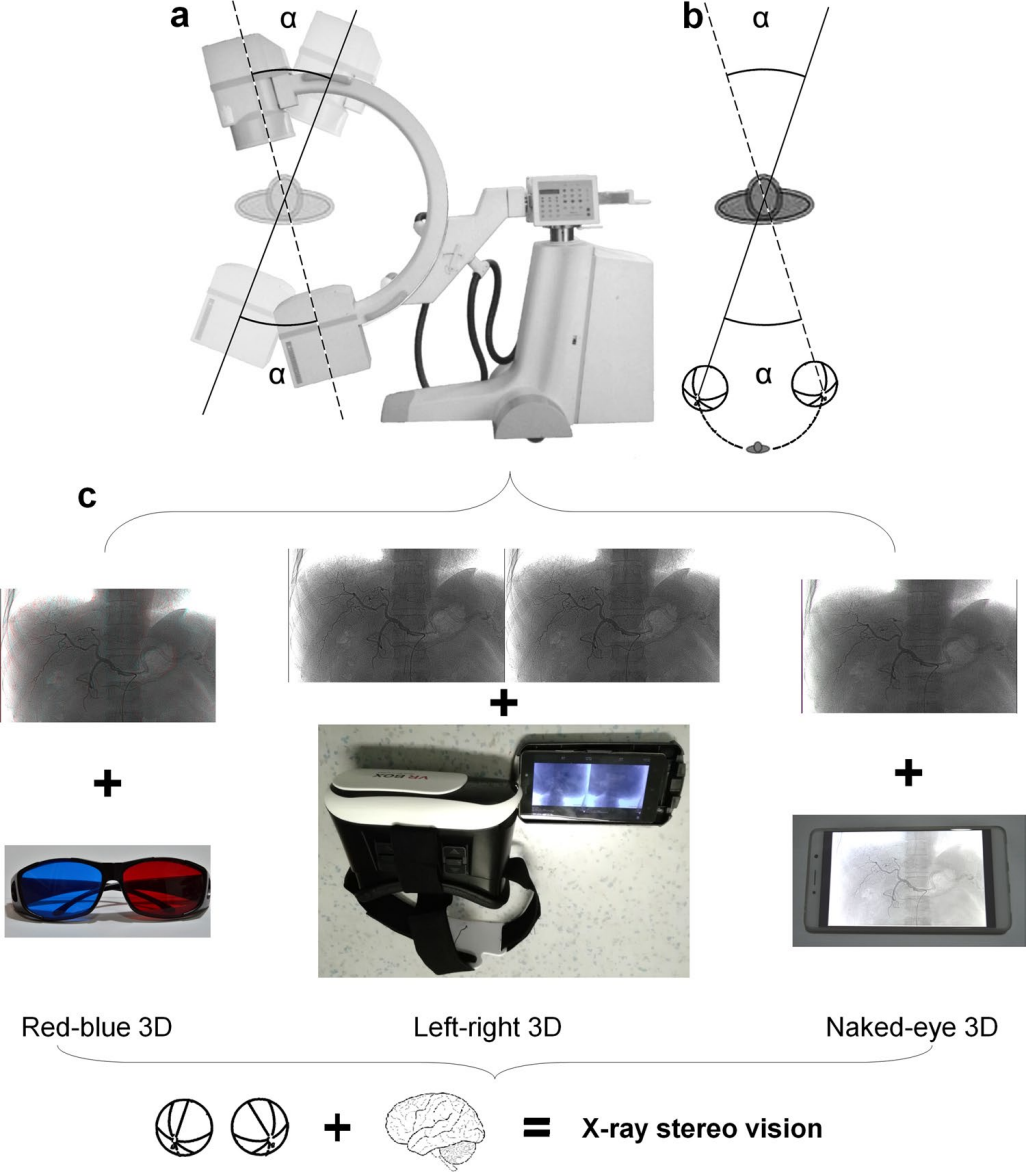
(**a**) Schematic diagram for the realization of stereo vision using X-ray fluoroscopy. (**b**) Schematic diagram for binocular observation. The α angle represents the difference between the angles at which two plain X-ray images or monocular views were captured. This angle was applied to construct two views with binocular disparity. (**c**) Three methods (red-blue 3D, left-right 3D and naked-eye 3D) used to realize the X-ray stereo vision.

The core principle of this technology involves simulating our visual process to simultaneously capture and display duplicate views from particular angles specific to humans. The human brain is equipped with powerful image processing capabilities that can generate 3D depth perception from two separate views with binocular disparity^11–14^. Therefore, we simply prepared two views from particular angles (Fig. 1a) and presented them to each eye (Fig. 1b). This angle (α angle) was applied to construct two views with binocular disparity. Observation with a VR headset, red-blue glasses or a naked-eye stereoscopic display (Fig. 1c) is sufficient for the brain to integrate the two images to generate X-ray stereo vision. This study focused on overcoming the lack of 3D depth perception in real-time guidance during interventional procedures.

## Results

### Realization of X-ray Stereo Vision during Interventional Procedures

**Figure 2:**
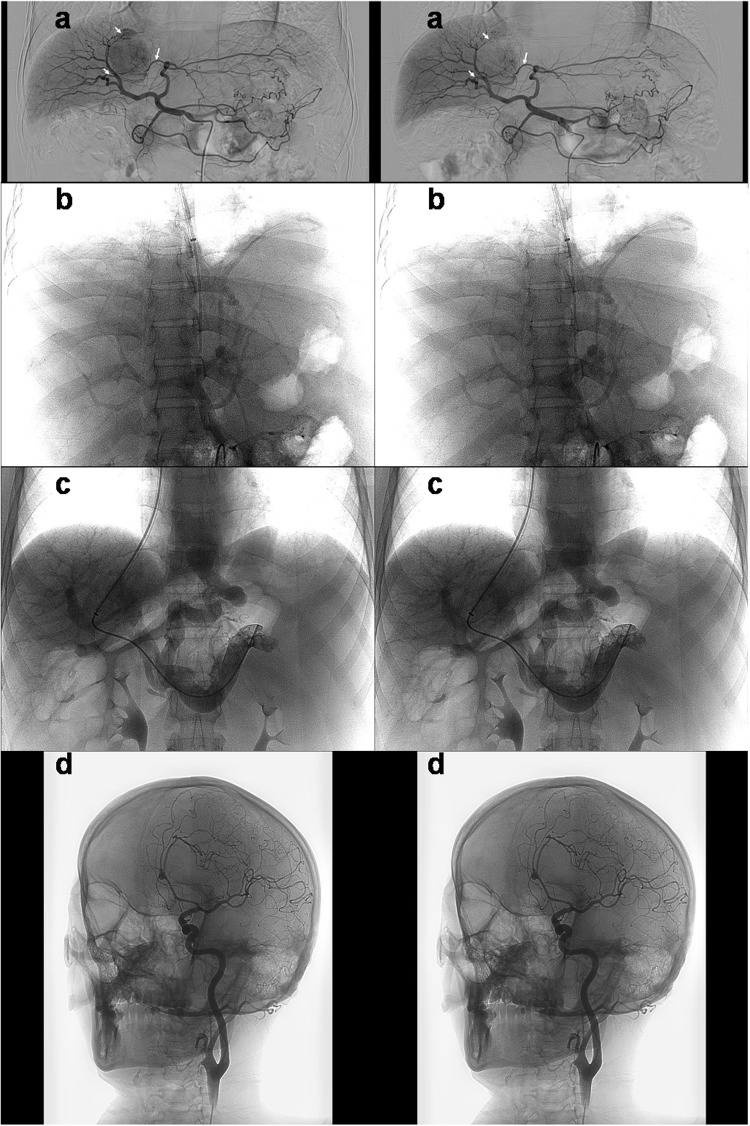
(**a**) Duplicate plain X-ray images from a patient with hepatic cancer during TACE were horizontally combined. The tumor’s supply vessels and the anteroposterior direction of overlapping vessels were clearly identified (the white arrows). Duplicate plain X-ray images of direct portography (**b**), indirect portography (**c**) and internal carotid angiography (**d**) with different α angles (RAO −16.0°/−18.0°, LAO 8.0°/10.0°, and LAO 22.8°/24.6°) were horizontally combined. Observation with VR glasses produced realistic stereo images of structures such as vessels, bones, angiographic guidewires and catheters.

**Figure 3:**
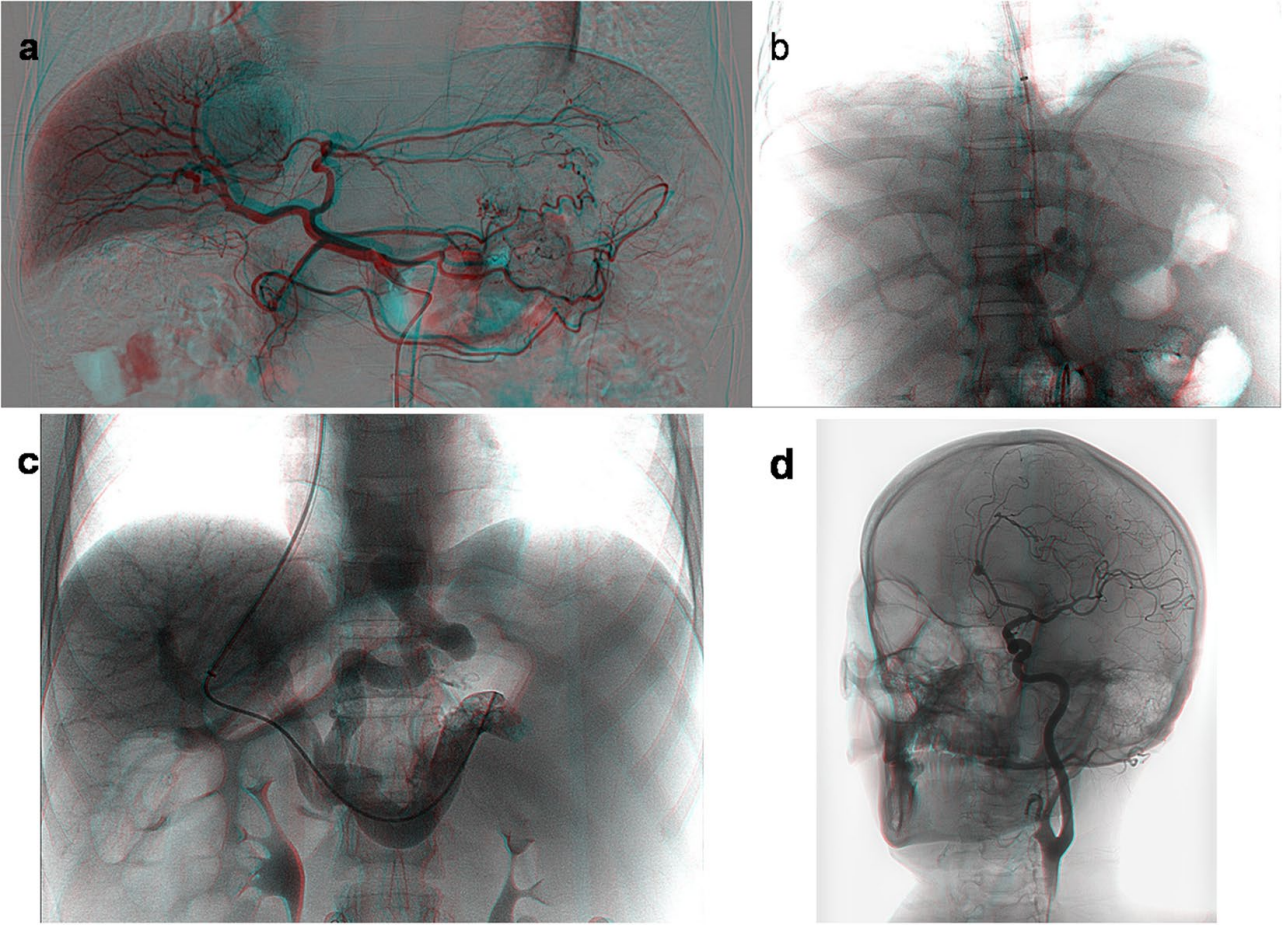
The above duplicated X-ray images (in Fig. 2) of angiography were translated into red-blue 3D images. Stereo images were viewed with a pair of red-blue 3D glasses (**a–d**).

During arterial-phase angiography^15^ (radiography performed for visualizing blood vessels while a contrast agent flow through arteries) (https://en.wikipedia.org/wiki/Angiography. Accessed July 23, 2018) of the celiac trunk in a patient with hepatic cancer during TACE, corresponding plain X-ray images with two different rotation angles were captured. These images were used as the left-eye and right-eye views. The views were horizontally merged into a left-right 3D image (shown in Fig. 2a) and translated into the red-blue 3D image shown in Fig. 3a. Using VR glasses or red-blue 3D glasses to observe the above images, the supply vessels and anteroposterior direction of overlapping vessels in the tumor can be quickly and easily visualized, and the spatial direction of the vessels and the surrounding anatomic structure can be more easily identified than when using plain X-ray images. Consequently, this technique was helpful for ultraselective TACE. In a second test, during portal-phase indirect/direct portography (radiography of the portal vein after the injection of a contrast agent) (https://en.wikipedia.org/wiki/Portography. Accessed July 23, 2018) in cirrhosis patients undergoing TIPS placement, corresponding plain X-ray images with two different rotation angles (right anterior oblique projection, RAO −18.0°/−16.0°; and left anterior oblique projection, LAO 8.0°/10.0°) (https://en.wikipedia.org/wiki/Projectional_radiography. Accessed July 23, 2018) were captured. The left and right images were horizontally merged (shown in Fig. 2b,c) and translated into the red-blue 3D image shown in Fig. 3b,c. In a third test, during left internal carotid angiography in a patient undergoing cerebrovascular intervention, corresponding plain X-ray images with two different rotation angles (LAO 22.8°/24.6°) were captured. The images were horizontally merged (shown in Fig. 2d) and translated into the red-blue 3D image shown in Fig. 3d. Using VR glasses or red-blue 3D glasses to observe the resulting image, a stereoscopic perspective of the arterial branches and their anteroposterior direction can be quickly visualized, and the spatial direction of the vessel and surrounding anatomic structure can be more easily identified than when using plain X-ray images.

In a subsequent test, the X-ray images described above were translated into interlaced X-ray stereoscopic images (Supplementary Figs S1–4) and displayed with a naked-eye 3D device (ZTE AXON 7 MAX C2017). This approach improved the observers’ spatial discrimination of anatomic structures. This interlaced stereoscopic imaging technology^16^ (a technique for respectively displaying odd and even rows of an image to the left and right eyes of the viewer to generate stereoscopic vision) (https://en.wikipedia.org/wiki/Integral_imaging. Accessed July 23, 2018) could be introduced to improve the accessibility of real-time X-ray stereo vision during interventional procedures.

### Effective Angle for Generating X-ray Stereo Vision

To avoid obvious double vision, the α angle was limited to less than 15°. To explore the effective angle between the pair of plain X-ray images, α angles (which represent the difference between the rotation angles in Fig. 1a,b) ranging from 0.7° to 15°, were tested in subsequent selective angiography of the carotid artery and celiac trunk. Pairs of duplicate X-ray images captured at different rotation angles were used to generate binocular vision with parallax (due to the different α angles) create the stereoscopic images. Several pairs of plain X-ray images from selective angiography were chosen to generate left-right stereoscopic images (Supplementary Figs S5–11 and S12–22). In the subsequent tests of stereo vision integration, the left-eye and right-eye views were accepted by the brain across a narrow range of α angles (carotid angiography: 1.8–9.1°, Supplementary Figs S5–9; selective angiography of the celiac trunk: 0.7–7.5°, Supplementary Figs S12–22). The optimal α angles for generating stereo vision are 1.8–3.6° (carotid angiography, Supplementary Figs S5,6) and 1.4–4.1° (selective angiography of the celiac trunk, Supplementary Figs S13–17). The corresponding range of optimal α angles mostly overlapped in the above angiography analyses, which shows that the brain can adapt to a variety of α angles within a certain range to achieve 3D depth perception under fluoroscopic vision. Choosing 1.8° as α angle, realistic stereo vision was repeatedly verified in the selective angiography (indirect/direct portography: Fig. 2b,c; internal/external carotid angiography: Fig. 2d and Supplementary Video S1; superior mesenteric arteriography: Supplementary Video S2). The results demonstrated that α angles generating binocular disparity may be acceptable within a certain range (approximately 1.4–4.1°). This phenomenon is similar to daily life in that our eyes can form stereo vision when viewing objects at a broad range of distances, generating binocular disparity. Furthermore, an individual possessing various pupil distances can adapt the sight diversity of the convergence angles of the eyes to achieve 3D depth perception. Therefore, the specific value for the optimal α angle is difficult to fix, but the effective range of α angles can be provided for reference. Similar to a binocular telescope with an adjustable pupil distance, a specific fluoroscope possessing biplane and dual X-ray sources with an adjustable convergence angle can simply and feasibly provide a certain range of α angles to generate individualized stereovision for users.

### Validating the Spatial Discrimination of X-ray Stereo Vision

**Figure 4:**
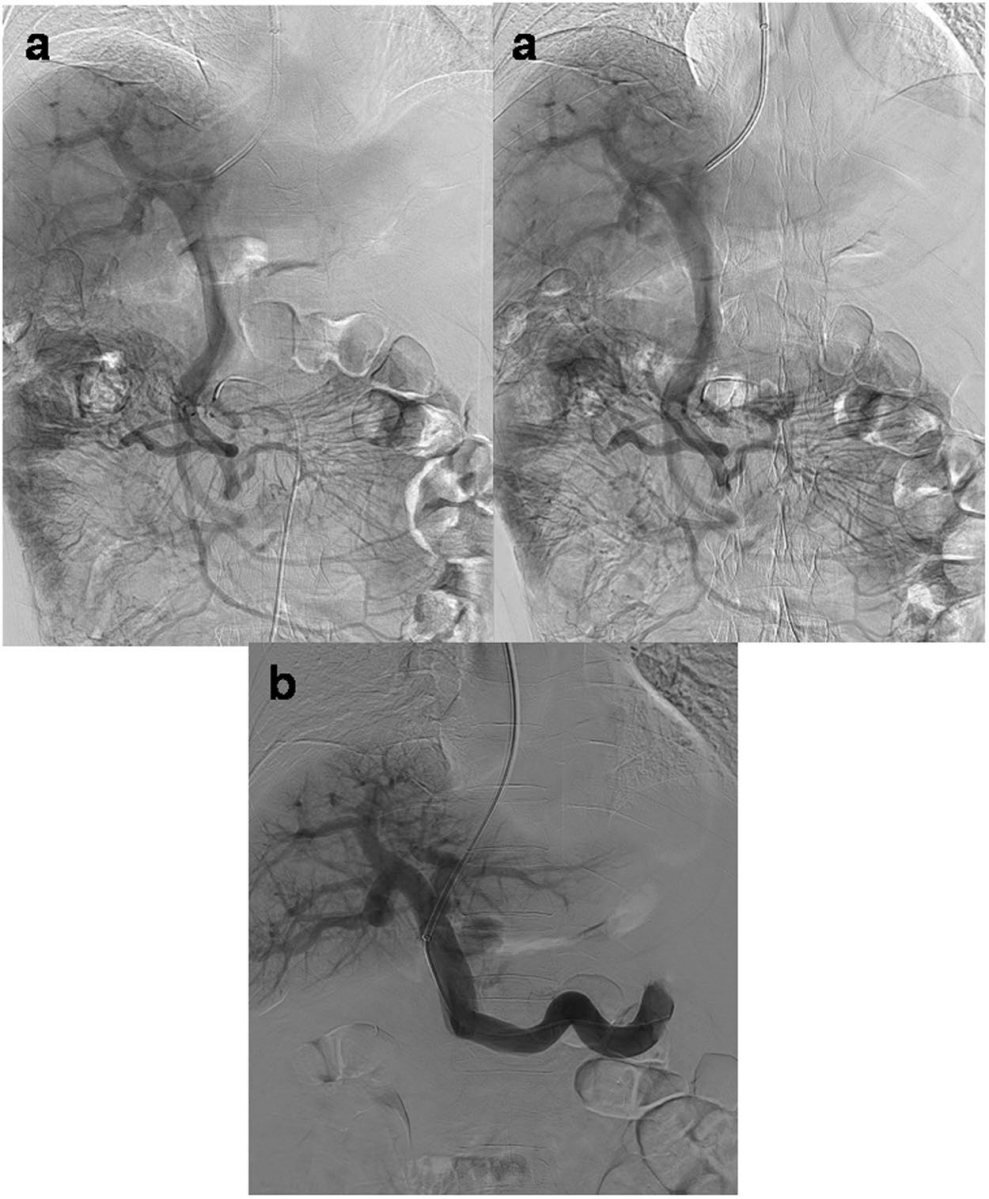
(**a**) In a 60-year-old man with TIPS placement, duplicate X-ray images of indirect portography at different rotation angles were horizontally combined to realize X-ray stereo vision to overcome repeated puncture failure. Observation with VR glasses produced stereo vision for easily identifying spatial relationships (the left portal vein was located at the left rear of the puncture needle). (**b**) After the puncture angle was adjusted toward the right rear, the puncture needle successfully entered the right portal vein. The subsequent portography confirmed that the needle had entered the right portal vein toward the right rear of the previous position.

The real-time X-ray stereo vision method was applied in a 60-year-old man undergoing TIPS placement for uncontrolled variceal bleeding. During the TIPS procedure, the puncture for establishing a tract from the hepatic vein to the portal vein failed more than three times. Two plain X-ray images of indirect portography at different rotation angles were captured and horizontally combined (Fig. 4a). Moreover, corresponding videos of indirect portography with duplicate and different rotation angles (RAO −0.1°/2.3°) were recorded and were considered as left-eye and right-eye movies. These movies were then translated into a left-right 3D movie (Supplementary Video S3), which was automatically translated into column-interlaced 3D video and displayed by a naked-eye 3D device (ZTE AXON 7 MAX C2017). This approach enabled the interventionalists to easily obtain X-ray stereo vision from the indirect portography. The spatial anatomical relationships could be clearly observed, revealing that the right portal vein was located to the right rear of the puncture needle. After the puncture angle was adjusted toward the right rear, the puncture needle successfully entered the left portal vein (Fig. 4b).

Six participants (2 interventionalists, 2 residents and 2 medical students) were invited to participate in a test to validate the clinical value of X-ray stereoscopic vision. In the primary test, the plain X-ray images from hepatic arteriography of hepatic cancer patients (31 cases) were delivered to the six participators to identify the supply vessels of a liver tumor and distinguish its anteroposterior direction. In the secondary test, the corresponding X-ray stereoscopic images with an optical binocular display (α angle: 2°) were presented to them again to identify the supply vessels of the liver tumor and distinguish its anteroposterior direction.

To validate the spatial discrimination capability of stereoscopic X-ray images, the six participants were invited to complete both primary and secondary tests, identifying the supply vessels and distinguishing the anteroposterior direction of overlapping vessels. Thirty-one pairs of primary and secondary tests were completed. The results showed that the improvement of spatial discrimination on the basis of X-ray stereoscopic vision was equal among the six participants (*P* = 0.735, Friedman Test, Table 1). The recognition rates from all six participants in the secondary test with stereoscopic X-ray images were significantly higher than those in the primary test with plain X-ray images (all *p* < 0.01, shown in Table 1). This finding demonstrated that the supply vessels of a hepatic tumor and its anteroposterior direction were easily confused when determined based on plain X-ray images but were accurately determined from stereoscopic X-ray images. Stereoscopic X-ray images provide valuable spatial resolution that assists interventionalists in discriminating the anteroposterior direction of overlapping tissues.

**Table 1:** Validating the spatial discrimination of X-ray stereoscopic vision during hepatic arteriography.

		Primary test		*P* value	Secondary test *vs*. Primary test			
		No	Yes		Good	Indistinct	Poor	*P* value
Interverntionalist 1					13	17	1	
Secondary test	No	4	1	0.002				
	Yes	13	13					
Interverntionalist 2					13	18	0	
Secondary test	No	2	0	<0.001				
	Yes	13	16					
Resident 1					16	12	3	
Secondary test	No	4	3	0.004				
	Yes	16	8					
Resident 2					18	11	2	
Secondary test	No	3	2	<0.001				
	Yes	18	8					
Medical student 1					19	9	3	
Secondary test	No	3	3	0.001				
	Yes	19	6					
Medical student 2					16	12	3	
Secondary test	No	5	3	0.004				
	Yes	16	7					
								0.735

## Discussion

In the field of interventional radiology, X-ray fluoroscopy can provide planar vision to operators for guiding and monitoring of procedures. However, this technique cannot directly render real-time stereo vision for interventionalists during operative procedures. Stereoscopic vision offers the perception of depth, which can provide large amounts of complex information^17^. Therefore, the difficulties associated with plain X-ray images may hinder doctors in identifying puncture angles and performing operations accurately and smoothly. X-ray images that provide binocular vision would significantly improve the perceptual separation of anatomic structures and background tissues, especially in interventional procedures involving complex or variant anatomy.

Stereo radiology was first proposed by Jarre *et al*.^18^. This approach is becoming an important topic with the potential to aid interventions. Drawing inspiration from stereo-imaging technology^10^, a simple method was established to achieve X-ray stereo vision via binocular disparity. In our tests, the identification of the supply vessels of the hepatic tumors and their anteroposterior direction, which is necessary for ultraselective TACE, was accurately and easily completed through the spatial discrimination provided by X-ray stereo vision. The results demonstrated that overlapping tissues can be clearly and easily distinguished via X-ray stereo vision without frequently changing positions.

TIPS placement is a technically challenging procedure for treating patients with cirrhosis and variceal bleeding^19,20^. The success of TIPS placement mainly depends on portal vein entry, for which identifying the anteroposterior direction and angle of the portal vein is very important. The value of X-ray stereo vision for identifying the anteroposterior direction and angle of the portal vein was confirmed in a challenging case of TIPS creation. The puncture failure was overcome through stereoscopic vision guidance (in Fig. 4a). The anteroposterior direction and angle of blood vessels can also be easily identified by this technique, which will be helpful in precise ultraselective procedures, especially in difficult cases with complex or variant anatomy. The advantages resulting from the spatial resolution of X-ray stereo vision may be valuable for reducing the difficulty of some operations, avoiding injury associated with unnecessary puncture, shortening the operative time, and decreasing the cumulative radiation exposure.

Given the restriction to monoplanar X-ray fluoroscopy in our center, generating real-time X-ray stereo vision by performing angiography twice with binocular disparity would be time consuming and complicated for operators. In addition, this approach would increase the risk of injury to patients associated with contrast agents and radiation exposure. These deficiencies can be easily overcome using biplanar X-ray fluoroscopy to simultaneously collect duplicate X-ray views from adjustable rotation angles. Wearing a VR box would be inconvenient for surgeons during operative procedures. However, the replacement of the VR box with polarized glasses and a polarized 3D display (or naked-eye 3D display^21–23^) would be straightforward and convenient for surgeons. Moreover, the simple law of perspective that objects appear larger when they are nearer to the observer is helpful for generating more realistic stereo vision (Supplementary Videos S4–5). If a point X-ray source can be introduced into the biplanar X-ray fluoroscopy system, the tissue shadow in the stereoscopic view will be larger when the tissue is near and smaller when it is further away, according to the distance ratio. Such devices can facilitate the generation of realistic, real-time X-ray stereo vision and can promote the implementation of some complex interventional procedures.

The creation of X-ray stereo vision may be beneficial not only in interventional procedures but also in overcoming some deficiencies of 2D radiography. The use of X-ray stereo vision in gastrointestinal radiology could provide additional stereoscopic information and fill some gaps, such as the spatial position and direction of the intestinal tract, to help clinicians achieve accurate diagnoses. Moreover, stereoscopic information about bony structures is necessary for some orthopedic procedures, such as femoral head arthroplasty and spine surgery. The stereoscopic information provided by X-ray stereo vision could help clinicians reduce the duration of these procedures.

It is easy for people create an impression that the generation of X-ray stereoscopic vision may cause an increased radiation dose during interventional procedures. The potential impact of the increase in dosage needs to be considered and controlled. For routine interventional procedures, conventional X-ray vision is recommended first. However, the operators may be confused when only conventional X-ray vision is used during certain complex interventional procedures for which stereoscopic vision is required. To compensate for the lack of 3D depth perception, they may repeatedly capture X-ray perspectives and use angiographic and cone-beam computed tomographic^24,25^ vision to accurately identify spatial information about target blood vessels and guide some difficult steps in interventional procedures. It is unavoidable that the exposure time to X-ray radiation will be prolonged to at least several times longer than the normal period. In fact, the exposure time is positively related to the dose during difficult steps in interventional procedures. In general, the total dosage of these steps may be increased at least several-fold compared with conventional times. In comparison to conventional X-ray vision, X-ray stereoscopic vision can provide a 3D perception of depth, which might be valuable in some cases, to allow interventionalists to naturally visualize complex spatial structures and easily perform difficult steps that require stereoscopic vision. The total exposure time in these steps may be greatly shortened when supported by 3D depth perception (as in the case shown in Fig. 4). Although the single dose involved in capturing X-ray stereoscopic vision is high, the total dose during the interventional procedure may be stable or even reduced. For achieving clinical aims, the radiation dose can be controlled to as low a level as possible because of the following factors. X-ray stereoscopic high-resolution vision shows only the area of focus where accurate operation needs to be performed, while conventional X-ray low-resolution vision shows the background area where the catheter status and bony landmarks need be monitored. This method for creating X-ray stereoscopic vision can only be used as an auxiliary to compensate for the weakness of conventional X-ray vision during key or difficult steps in complex interventional procedures, which would be useful for eliminating the additional impact of the increased dose associated with capturing the X-ray stereoscopic vision. Therefore, whether the application of X-ray stereoscopic imaging increases the total exposure dose or not is still unclear.

With the development of computer science, MR, VR and AR have been proposed and introduced to solve clinical problems. VR technology has been used to relieve mental illness^26^, to assistant in rehabilitation therapy^27^, to train surgeons and interventionalists in clinical skills^28–31^ and so on. VR shows a simulated environment without the real-world environment. To implement perfect integration with the real–world environment for medical educational training^4,5^, stroke rehabilitation^32^, psychotherapy^6,33^ and so on, VR has been alternated with AR and MR which merge the real and virtual environments. Moreover, it is clear that AR and computer-mediated reality are very helpful for navigation in medical applications (e.g., orthopedic surgery^7–9^, interventional procedures^9,34–36^). Complex interventional procedures with a high requirement for spatial information may be difficult and time consuming to perform under fluoroscopic guidance without 3D depth perception. Many methods using AR and computer-mediated navigation were developed to guide these complex interventional procedures. In comparison to computer-assisted navigation sing CT scanning, navigation using X-ray fluoroscopy can easily decreased the radiation dose involved^37^ and provide real-time guidance for doctors^38^ but diminishes 3D depth perception. The X-ray stereoscopic imaging associated fluoroscopic radiation dose and 4D spatiotemporal visualization (3D volume plus time) exhibit the above advantages. X-ray stereoscopic imaging can be a valuable and simple method for providing real-time guidance with 3D depth perception. AR or computer-mediated reality technology can produce a new visualization where real and virtual vision are merged in real time. With its complementary advantages, X-ray stereoscopic imaging will play a useful role in capturing the real-word environment in a 3D-plus-time model for construction or destruction of the overlaid sensory information during AR, MR or computer-mediated navigation.

Many three-dimensional (3D) roadmapping and navigational tools are already available for use in interventional radiology^39–41^. These technologies are widely applied in many interventional procedures. Their value is unquestionable, but some deficiencies remain. For example, the patient’s position must be absolutely fixed. For interventionalists, real-time and realistic visualization is difficult to achieve with the reconstructed 3D images provided by existing technologies. X-ray stereoscopic vision on the basis of binocular disparity may be a promising technology for overcoming the above problems. It will be realized in the orthopedic fixation surgery that the surgeons possessing 3D depth perception might easily insert the orthopedic coil screws into the holes of a bone plate under the real-time guidance by X-ray stereoscopic fluoroscopy.

This study provides a straightforward and simple method for creating real-time X-ray stereo vision. This technique can help unify stereo vision and time in X-ray fluoroscopy to successfully achieve four-dimensional (3D plus time spatiotemporal visualization). This method will provide greater convenience to interventionalists to implement more complex operations and will have a profound impact on the precision of interventional medicine. Thus, this is a promising and valuable approach that can be applied in a growing number of fields that utilize medical imaging.

## Methods

### Ethics statement

This study was approved by the Ethics Committee Board of West China Hospital. All methods were performed in accordance with relevant guidelines and regulations. Written informed consent was obtained from each participant.

### Patients

Four cirrhosis patients receiving TIPSs for uncontrollable bleeding, one hepatic cancer patient undergoing TACE, two patients with cerebrovascular disease undergoing carotid angiography and one patient with a ruptured splenic artery aneurysm undergoing interventional embolization were recruited between September 2016 and January 2017. Their X-ray images were collected and utilized to establish the model for achieving X-ray stereo vision.

### Selective Angiography

Omnipaque injection (Iohexol Injection, 300 mg/ml, GE HEALTHCARE) was used as an intravenous contrast medium in the below procedures. The C-arm system (UNIQ Clarity FD20, Philips Medical Systems, Royal Philips, Amsterdam, Netherlands) was applied in the following interventional procedures. The following procedures for TIPS placement were applied. After local anesthesia was achieved and the right internal jugular vein was punctured, a Rosch-Uchida Transjugular Liver Access Set (Rups100, Cook, Bloomington, IN) was introduced into the inferior vena cava. After catheterization of the hepatic vein, the puncture site and penetration angle were estimated in accordance with hepatic venography and preoperative three-dimensional CT angiography. A long curved needle was then passed through the hepatic vein and the liver parenchyma into an intrahepatic branch of the portal vein. After portal vein entry was confirmed, the intrahepatic tract was accessed through the sheath using an angled hydrophilic guidewire (Terumo Company, Fijinomiya, Japan), and a 5-F catheter (Terumo Company, Fijinomiya, Japan) was advanced into the superior mesenteric or splenic vein. Portography was performed with the 5-F catheter placed in the portal region. In patients with difficult portal vein entry during TIPS creation, indirect portography was used. Superior mesenteric angiography and indirect portography were achieved in arterial- and portal-phase superior mesenteric angiography, respectively, which were performed with the catheter placed in the superior mesenteric artery. Selective angiography was achieved in arterial-phase carotid angiography, which was performed with the catheter placed in the internal or external carotid artery.

### Real-time X-ray Stereo Vision

X-ray images or videos of selective angiography were captured with the same operating position and parameter settings, except for different rotation angles (angle difference ranging from 0.7° to 12.8°), using a C-arm system (UNIQ Clarity FD20, Philips Medical Systems). The corresponding images with two different rotation angles were used as left-eye and right-eye views and were horizontally merged into single left-right 3D images. Stereo vision was achieved using VR glasses (a simple VR box, Fig. 1c) to observe the merged images. Alternatively, corresponding images with two different rotation angles were translated into single red-blue 3D images, and red-blue glasses (Fig. 1c) were used to observe the edited images, generating stereo vision.

To improve the accessibility of real-time X-ray stereo vision during interventional procedures, interlaced 3D imaging technology for a naked-eye 3D display was introduced by translating left-eye and right-eye views into single column-interlaced 3D images (1920 pixels in width) using StereoPhoto Maker 4.52. Using a naked-eye 3D device (ZTE AXON 7 MAX C2017, a mobile phone equipped with a 1920*1080 pixels naked-eye 3D display screen, Fig. 1c) to display these column-interlaced images, the operators can easily access X-ray stereo vision during interventional procedures.

### Exploring the Effective Angle for Generating Binocular Disparity

To explore the optimal α angle, X-ray images (a cerebral prop scan and abdominal scan were completed in 2 seconds; scan range: LAO 90° - RAO 90° or more) of selective angiography (internal/external carotid angiography, 1.8°/frame; celiac trunk, 0.7°/frame) were obtained and recorded. To avoid double vision, the α angle was limited to less than 15°. Any pair of images with different α angles (the difference between a pair of rotation angles, ranged from 0.7° to 12.8°, as shown in Fig. 1a,b) was chosen to generate binocular disparity and merged into a left-right 3D image (Supplementary Figs S5–22). Based on the use of VR glasses to observe these left-right 3D images, the five authors were invited to judge the acceptable and optimal ranges of α angles for generating X-ray stereoscopic vision from these edited images. The ranges of α angles for achieving X-ray stereoscopic vision accepted by over half of the authors (3/5) and all five authors (5/5) were defined as the acceptable and optimal values, respectively. The requirements regarding body position, projection angle, the size of the fluoroscopic area, etc., are diverse among the different kinds of radiographic procedures. These variables may cause deviation of the optimal α angle. The effective angle identified from a certain radiographic procedure may be applied in a specific situation. Thus, the ranges of effective angles was modified according to the different medical procedures.

### Validating the Spatial Discrimination of X-ray Stereo Vision

Thirty-one patients with hepatic cancer hospitalized for TACE were recruited. Hepatic arteriography was performed twice (α angle: 2°). The anteroposterior direction of the supply vessels of hepatic tumors with overlapping tissues was difficult to distinguish based on these plain X-ray images, but was finally identified and validated by interventionalists according to results obtained from previous CT angiography (CTA) and multiple radiography. To validate the spatial discrimination capability of X-ray stereo vision, six participants (2 interventionalists, 2 residents and 2 medical students) were invited to complete a pair of tests. In the primary test, the plain X-ray images from these angiograms were delivered to the  six participators  to identify the supply vessels of a liver tumor and distinguish its anteroposterior direction. In the secondary test, the corresponding stereoscopic X-ray images with optical binocular display (α angle: 2°) were presented to them again to identify the supply vessels of the liver tumor and distinguish its anteroposterior direction. During the tests, the plain X-ray (1920 pixels in width) and stereoscopic (column-interlaced 3D mode, 1920 pixels in width) images were display via the naked-eye 3D device (ZTE AXON 7 MAX C2017). Thirty-one pairs of primary and secondary tests were completed by every participator. If the participant’s answer in the same case in the secondary test was better than that in the primary test, the result was marked as “good”. If the participant’s answer in the same case in the secondary test was equal to that in the primary test, the result was marked as “indistinct”. If the participant’s answer in the same case in the secondary test was worse than that in the primary test, the result was marked as “poor”. The above judgment was performed in each case for every participator. The Friedman test was used to determine whether the improvement of spatial discrimination on the basis of X-ray stereoscopic vision was equal among the six participators. McNemar’s test was employed to determine whether the frequency of correct cases for a given participator in the primary and secondary tests was equal. McNemar’s test was performed for the results from every participator. All *p* values were two sided, and significance was assumed at the 5% level. All calculations were performed with Excel 2010 (Microsoft Corporation, Seattle, WA, USA), and statistical tests were calculated using SPSS version 15.0 (SPSS, Chicago, IL, USA).

### Software

The Philips DICOM Viewer (Version R3.0 SP3) was used to display the original plain X-ray images. The plain X-ray images in each pair were horizontally merged into left-right 3D images and translated into 3D red-blue and column-interlaced 3D images using StereoPhoto Maker (Version 4.52). The plain X-ray movie pairs were translated into left-right 3D movies (Supplementary Videos S1–3).

## Electronic supplementary material

Supplementary information

Supplementary Video S1

Supplementary Video S2

Supplementary Video S3

Supplementary Video S4

Supplementary Video S5
