# Supplementary material for: Realization of real-time X-ray stereoscopic vision during interventional procedures
Source: Sci Rep. 2018 Oct 26;8:15852. doi: 10.1038/s41598-018-34153-9 (PMC6203764; doi:10.1038/s41598-018-34153-9)
Supplement: Supplementary file 1 — Supplementary information [file 41598_2018_34153_MOESM1_ESM.pdf]

Realization of real-time X-ray stereoscopic vision during interventional procedures

Kai Deng[1,+], Bo Wei[1,+], Mo Chen[2], Zhiyin Huang[1] and Hao Wu[1,*]

[1] Department of Gastroenterology, West China Hospital, Sichuan University, 37 Guoxue Lane, 610041, Chengdu, Sichuan Province, China

[2] Department of Gastroenterology, Tibetan Chengdu Branch Hospital of West China Hospital, Sichuan University, No. 20, Heng Street, Ximian Bridge, 610041, Chengdu, Sichuan Province, China

[*] Corresponding author: 594264513@qq.com

[+] These authors contributed equally to this work.

Competing Interests: The authors declare no competing interests.

Supplementary Information List

Supplementary Figures

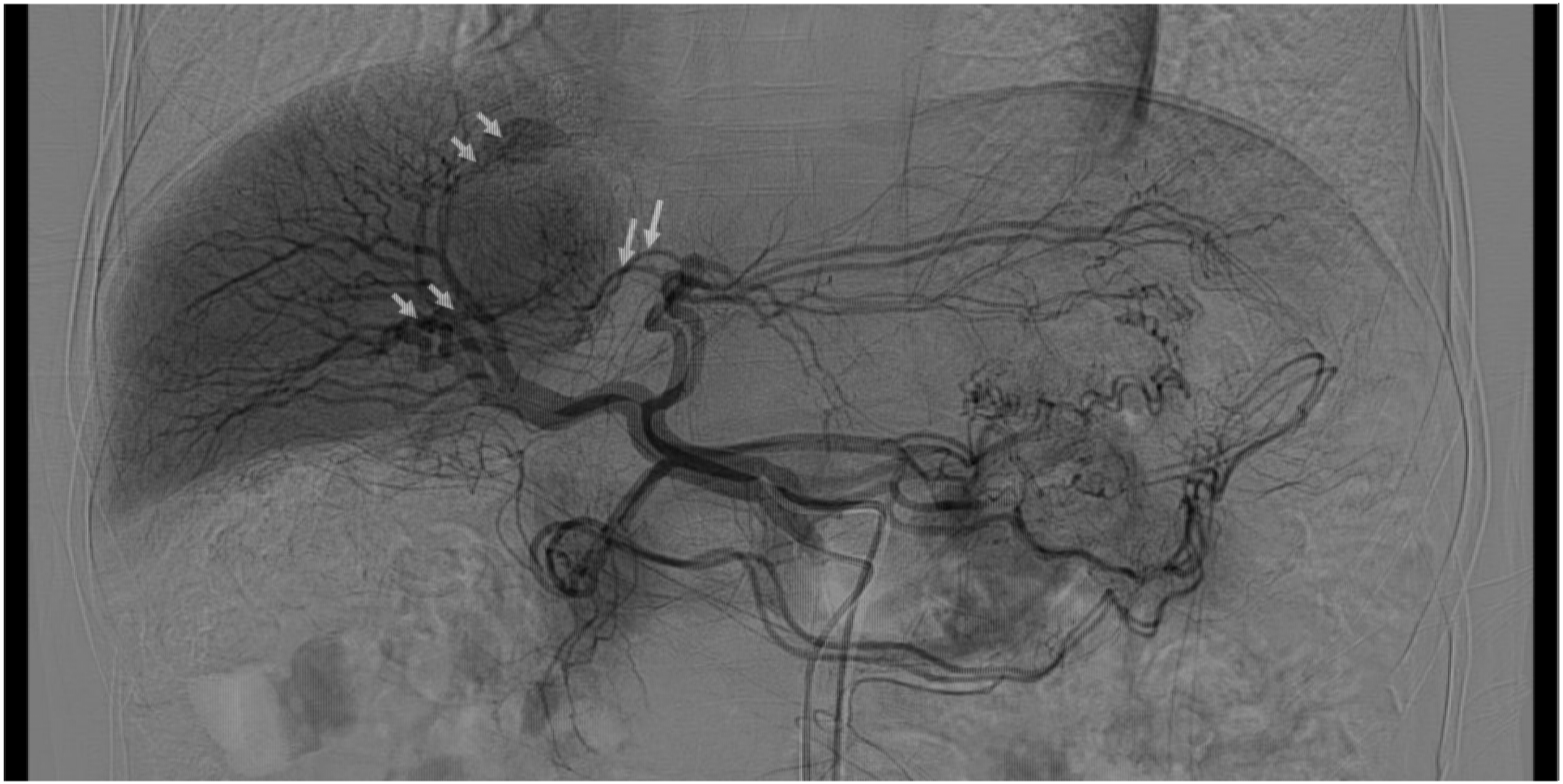

Supplementary Figure S1. The X-ray image in Fig. 2A was translated into a column-interlaced 3D image (1920 pixels in width).

X-ray stereo vision could be easily achieved via a naked-eye 3D device (ZTE AXON 7 MAX C2017).

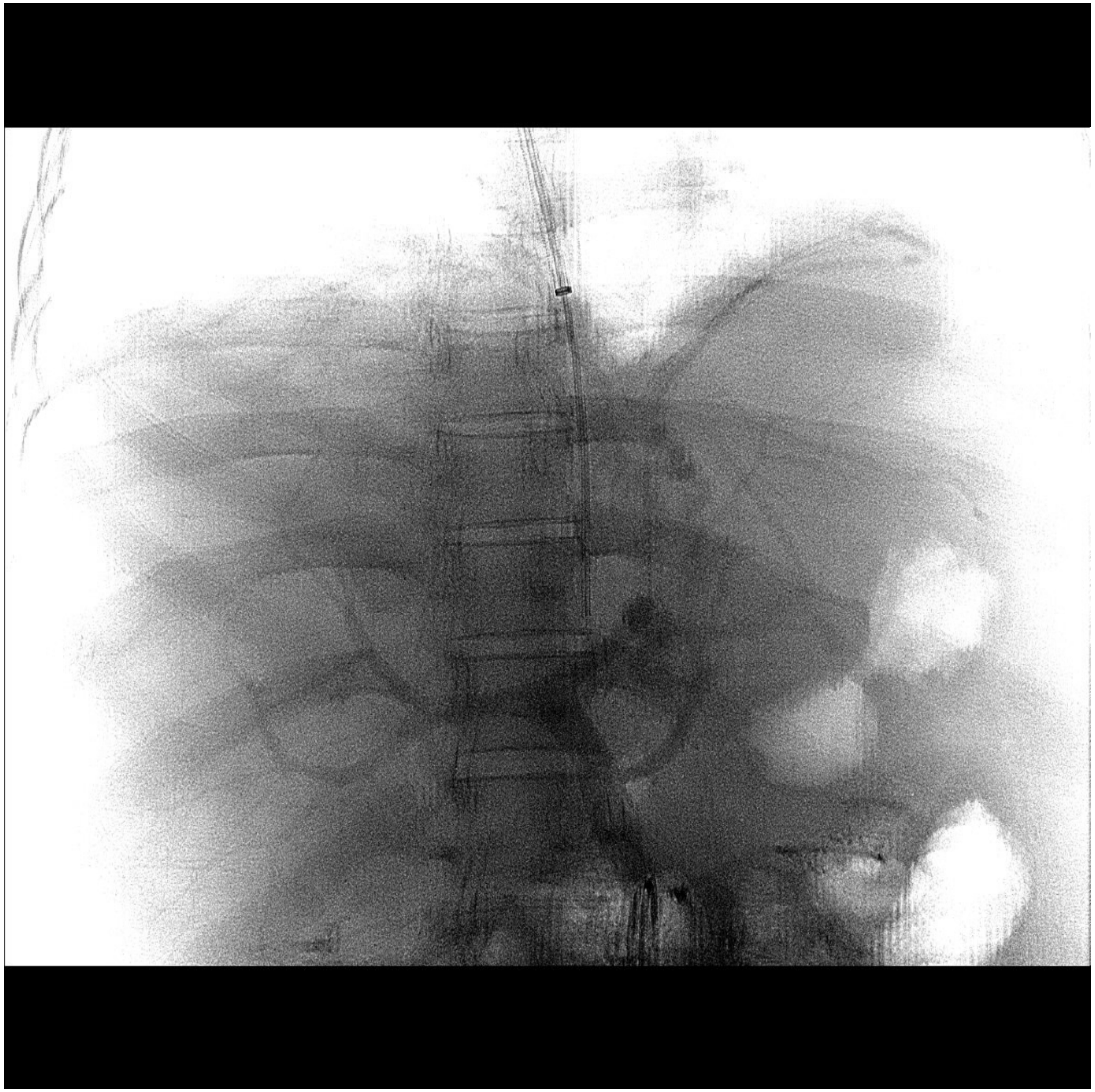

Supplementary Figure S2. The X-ray image in Fig. 2B was translated into a column-interlaced 3D image (1920 pixels in width). X-ray stereo vision could be easily achieved via a naked-eye 3D device (ZTE AXON 7 MAX C2017).

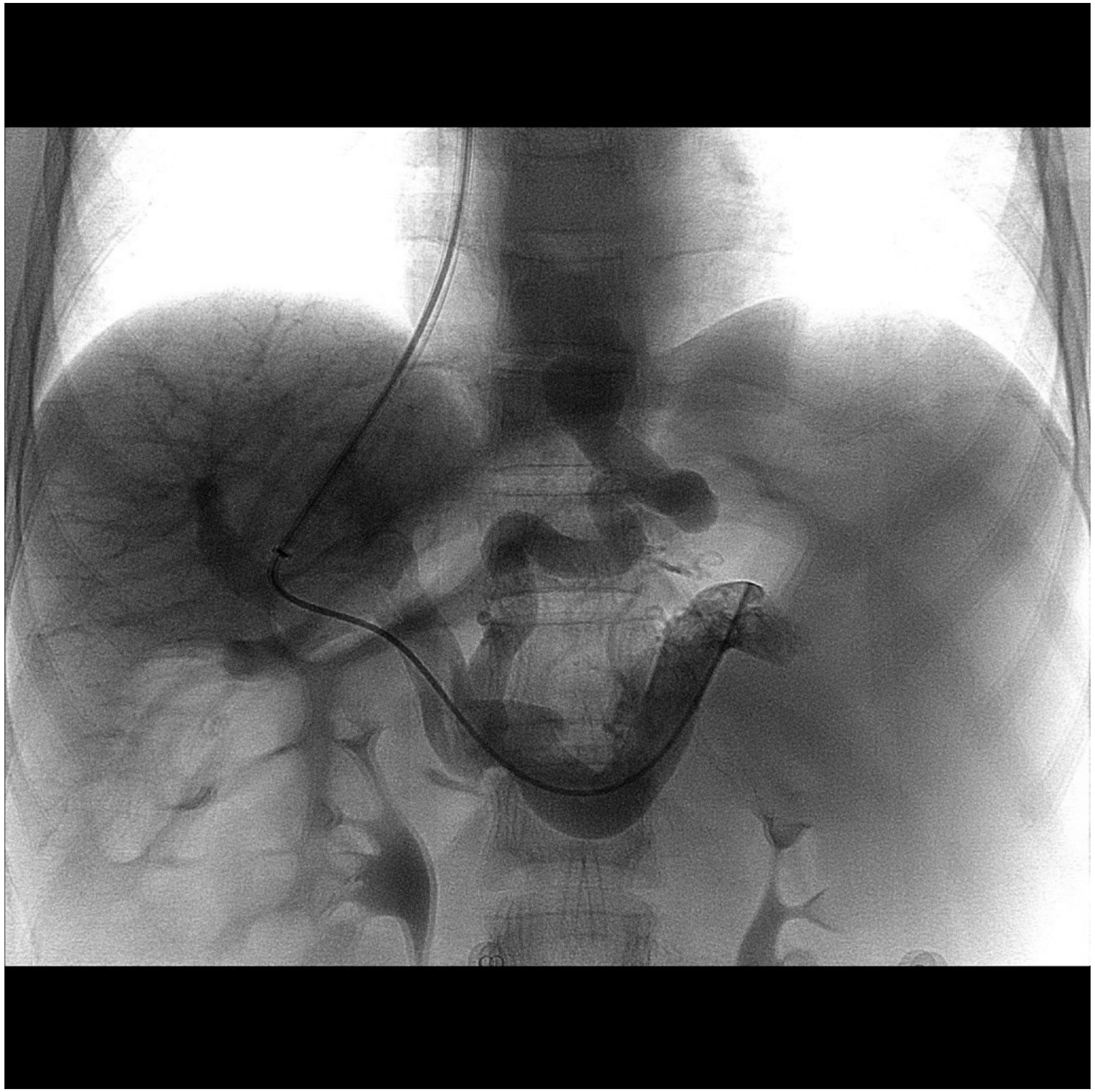

Supplementary Figure S3. The X-ray image in Fig. 2C was translated into a column-interlaced 3D image (1920 pixels in width). X-ray stereo vision could be easily achieved via a naked-eye 3D device (ZTE AXON 7 MAX C2017).

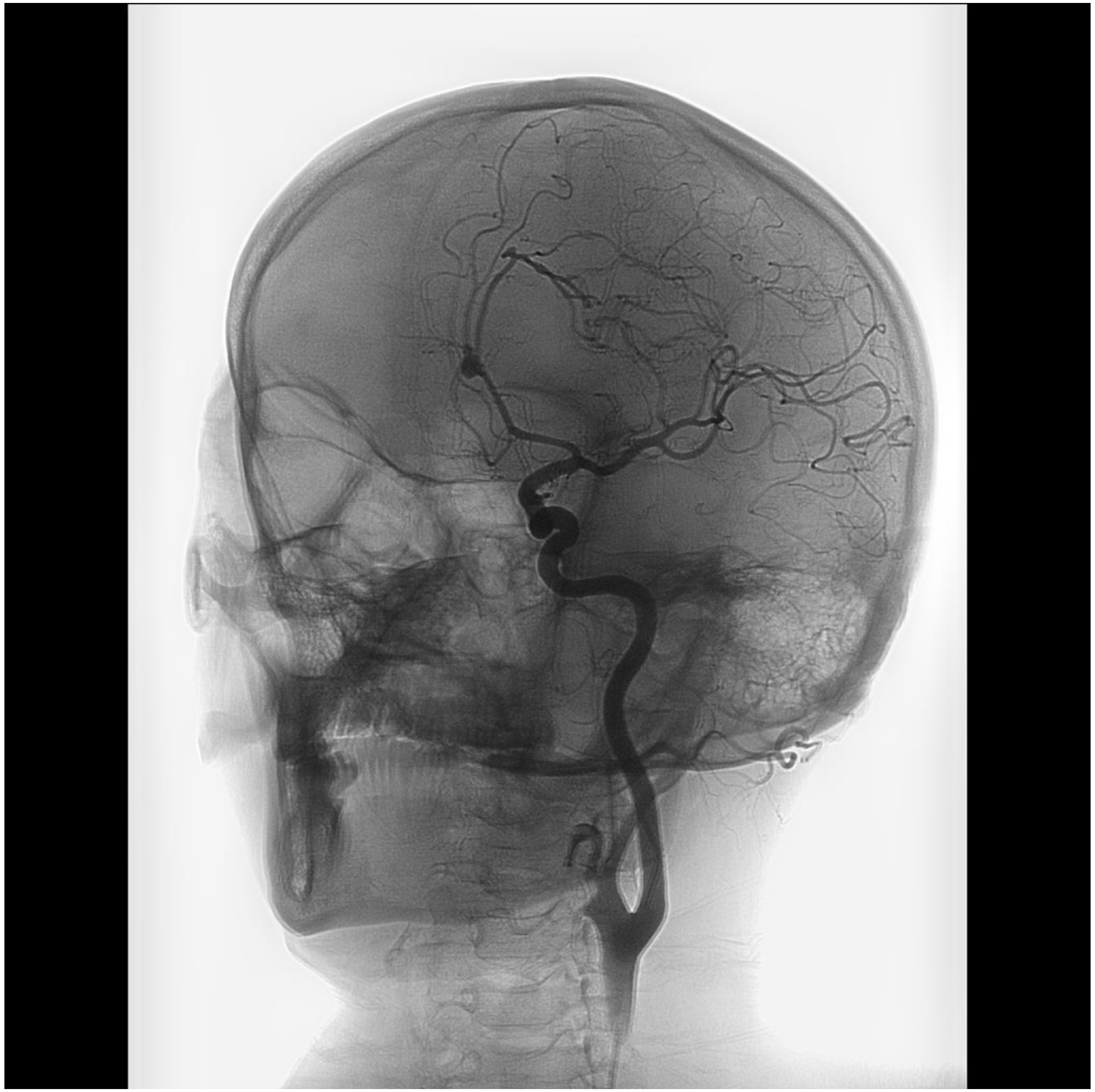

Supplementary Figure S4. The X-ray image in Fig. 2D was translated into a column-interlaced 3D image (1920 pixels in width). X-ray stereo vision could be easily achieved via a naked-eye 3D device (ZTE AXON 7 MAX C2017).

Case One

A patient with left internal carotid artery stenosis received interventional treatment. A cerebral prop scan (1.8° per frame) was performed and recorded during carotid angiography. To explore the acceptable range of α angles, two plain X-ray images with a certain angle were horizontally combined. Examples of the left-right 3D images are shown in Supplementary Figs. 5-11. These images were evaluated by five individuals to determine the acceptable range of α angles for generating stereo vision. The acceptable range of α angles was 1.8°-9.1°. The optimal range of α angles was 1.8°-3.6°. A left-right 3D video with an optimal angle (1.8°) is presented in Supplementary Video S1.

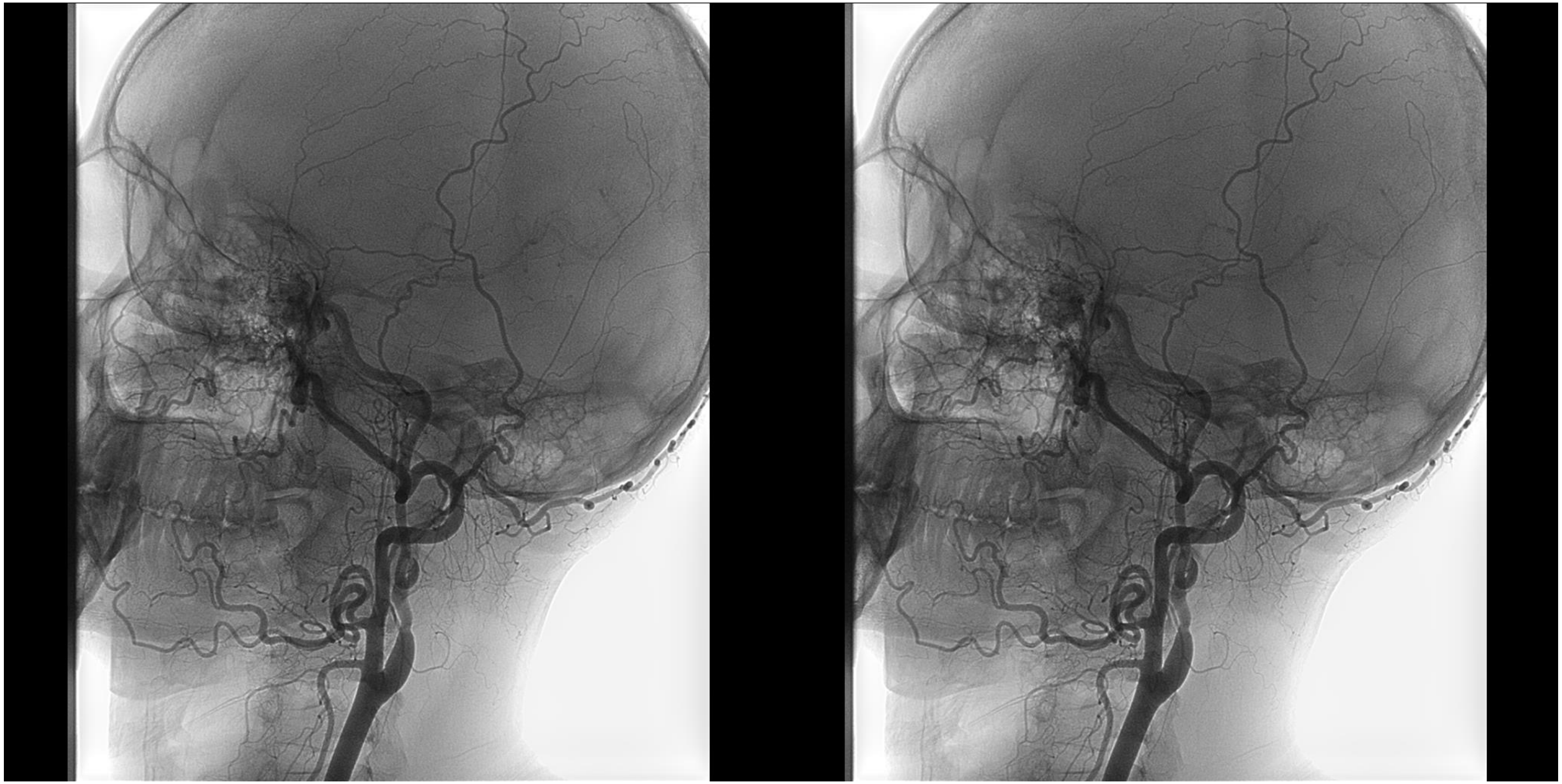

Supplementary Figure S5. Duplicate plain X-ray images of carotid angiography with different rotation angles (LAO 46.6°/44.8°) were horizontally combined. The α angle between the left and right X-ray images is 1.8°.

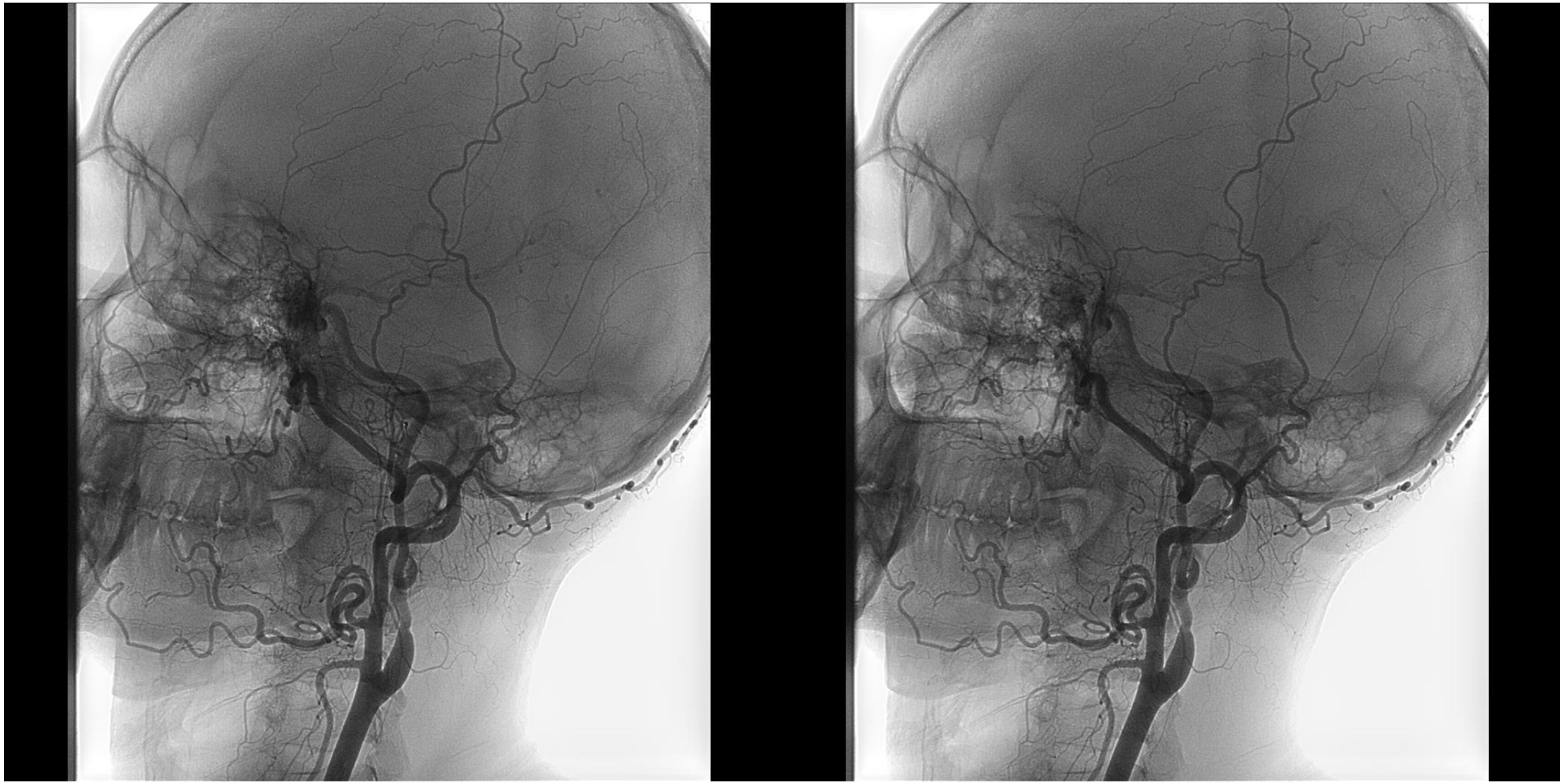

Supplementary Figure S6. Duplicate plain X-ray images of carotid angiography with different rotation angles (LAO 48.4°/44.8°) were horizontally combined. The α angle between the left and right X-ray images is 3.6°.

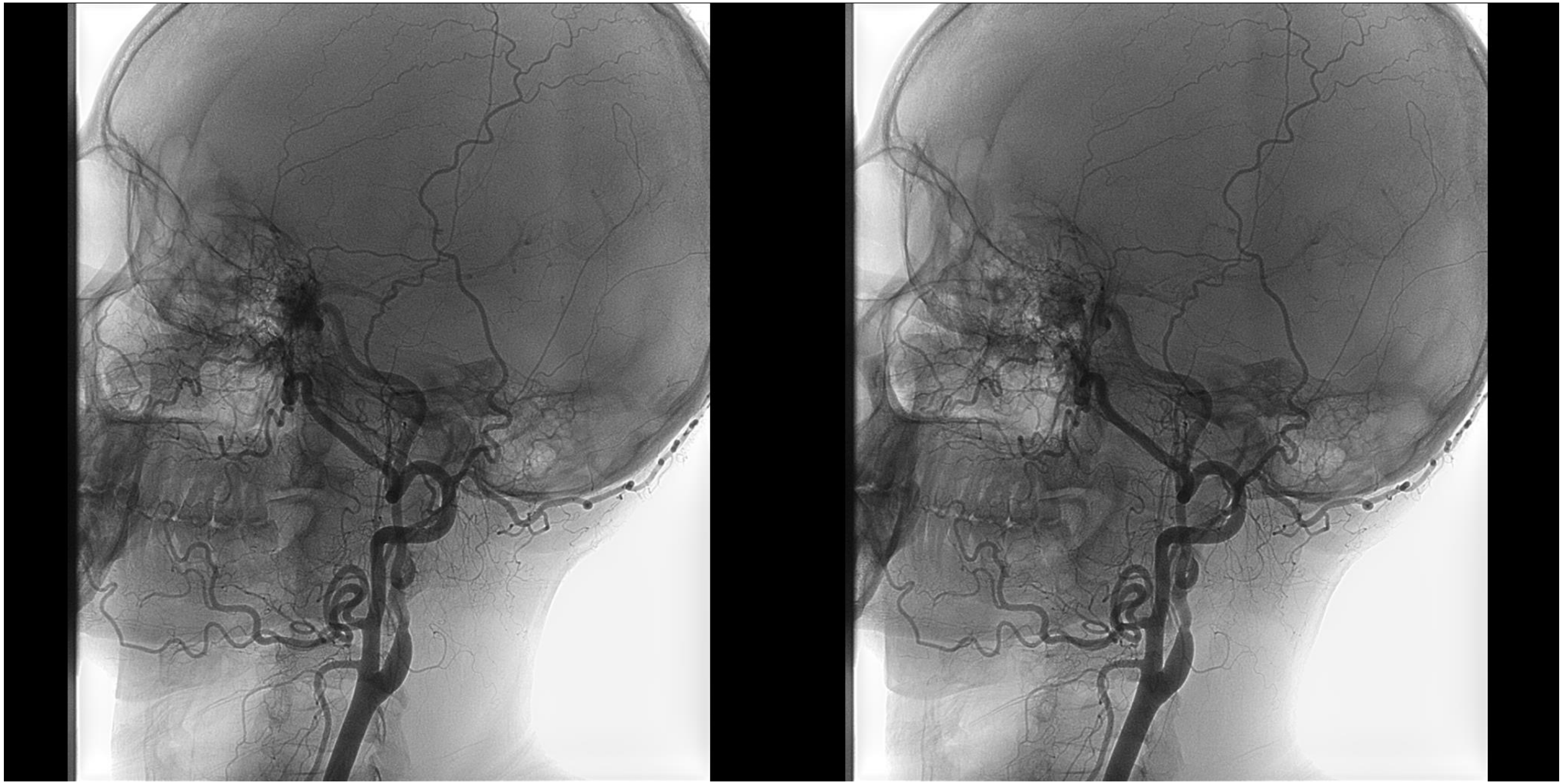

Supplementary Figure S7. Duplicate plain X-ray images of carotid angiography with different rotation angles (LAO 50.3°/44.8°) were horizontally combined. The α angle between the left and right X-ray images is 5.5°.

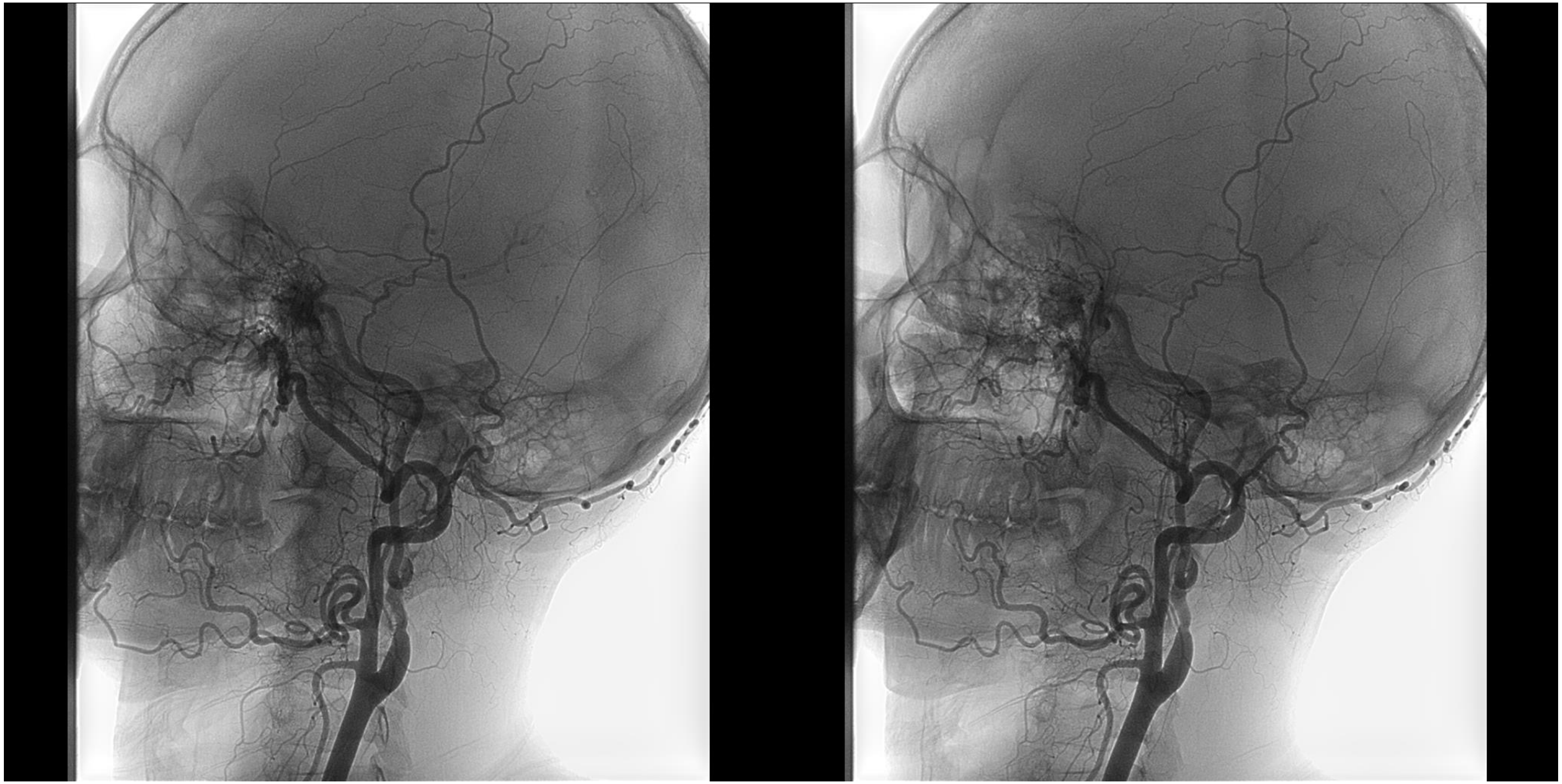

Supplementary Figure S8. Duplicate plain X-ray images of carotid angiography with different rotation angles (LAO 52.1°/44.8°) were horizontally combined. The α angle between the left and right X-ray images is 7.3°.

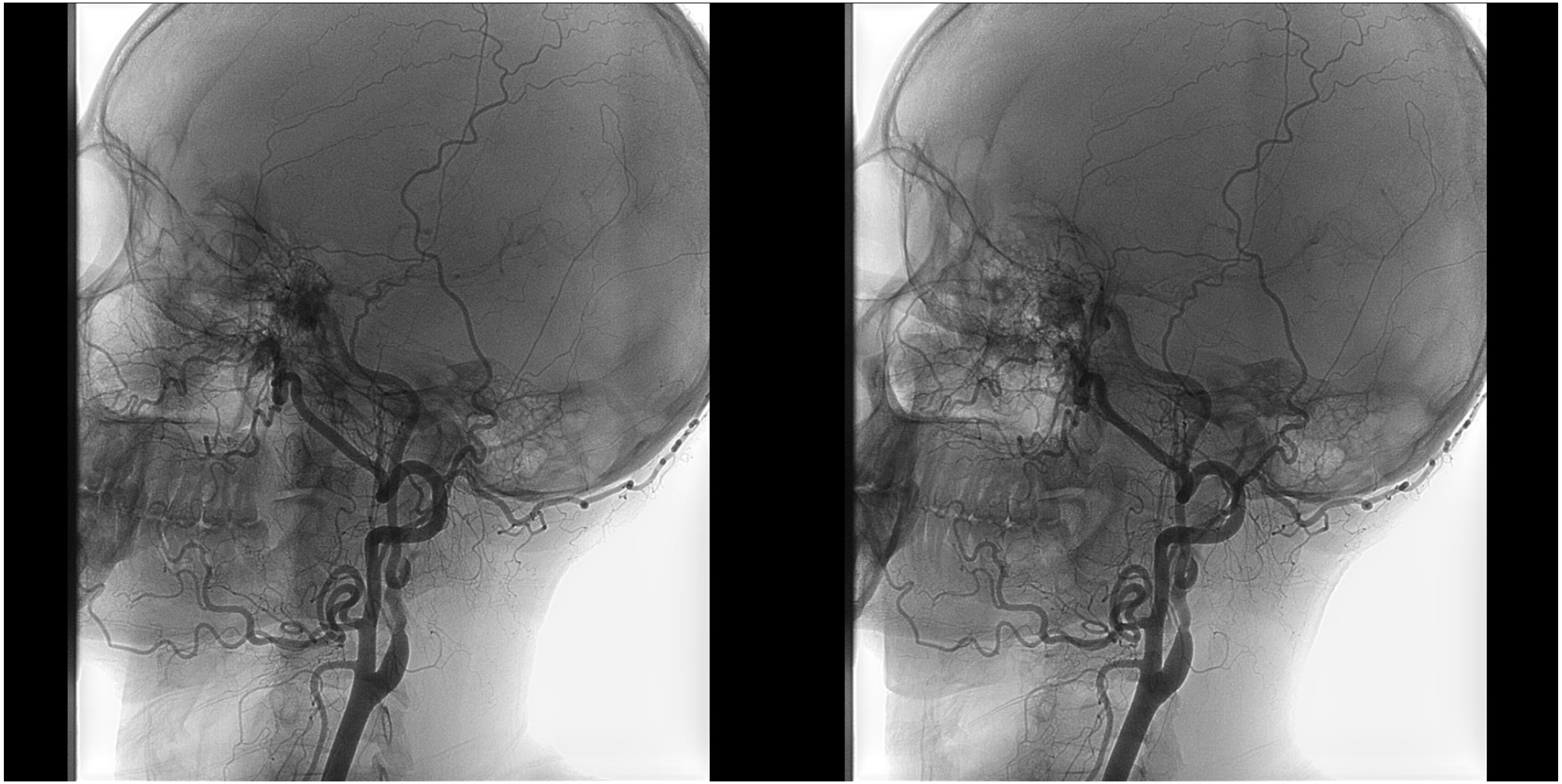

Supplementary Figure S9. Duplicate plain X-ray images of carotid angiography with different rotation angles (LAO 53.9°/44.8°) were horizontally combined. The α angle between the left and right X-ray images is 9.1°.

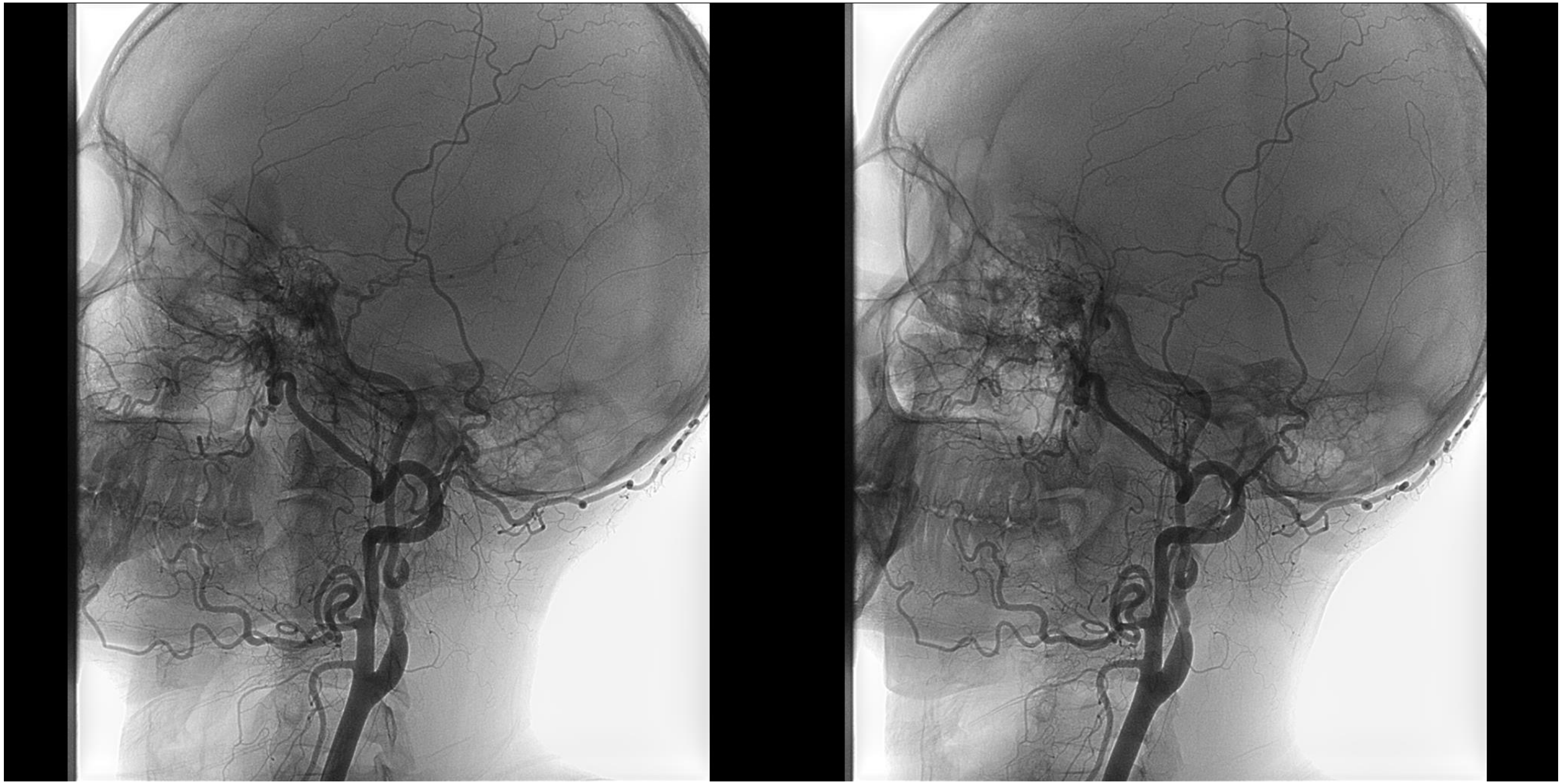

Supplementary Figure S10. Duplicate plain X-ray images of carotid angiography with different rotation angles (LAO 55.8°/44.8°) were horizontally combined. The α angle between the left and right X-ray images is 11°.

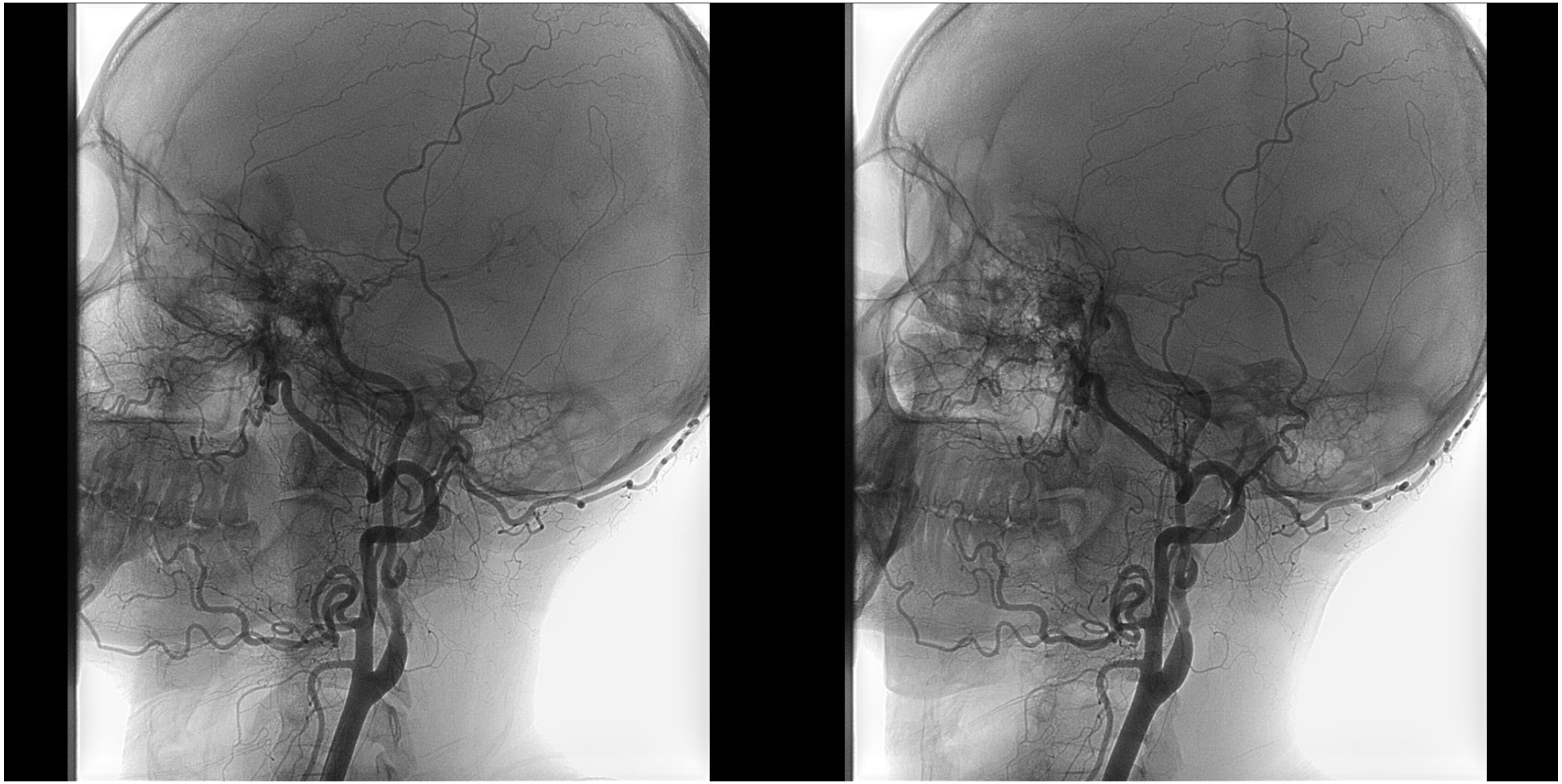

Supplementary Figure S11. Duplicate plain X-ray images of carotid angiography with different rotation angles (LAO 57.6°/44.8°) were horizontally combined. The α angle between the left and right X-ray images is 12.8°.

Case Two

A patient with hepatic cancer received transcatheter arterial chemoembolization (TACE). An abdominal roll scan (1.8° per frame) was performed and recorded during selective angiography of the celiac trunk. To explore the acceptable range of α angles, two plain X-ray images with a certain angle were horizontally combined. Examples of the left and right images are shown in Supplementary Figs. 8-18. These images were evaluated by five individuals to determine the acceptable range of α angles for generating stereo vision. The acceptable range of α angles for generating stereo vision from these images was 0.7°-7.5°. The optimal range of α angles for generating stereo vision was 1.4°-4.1°.

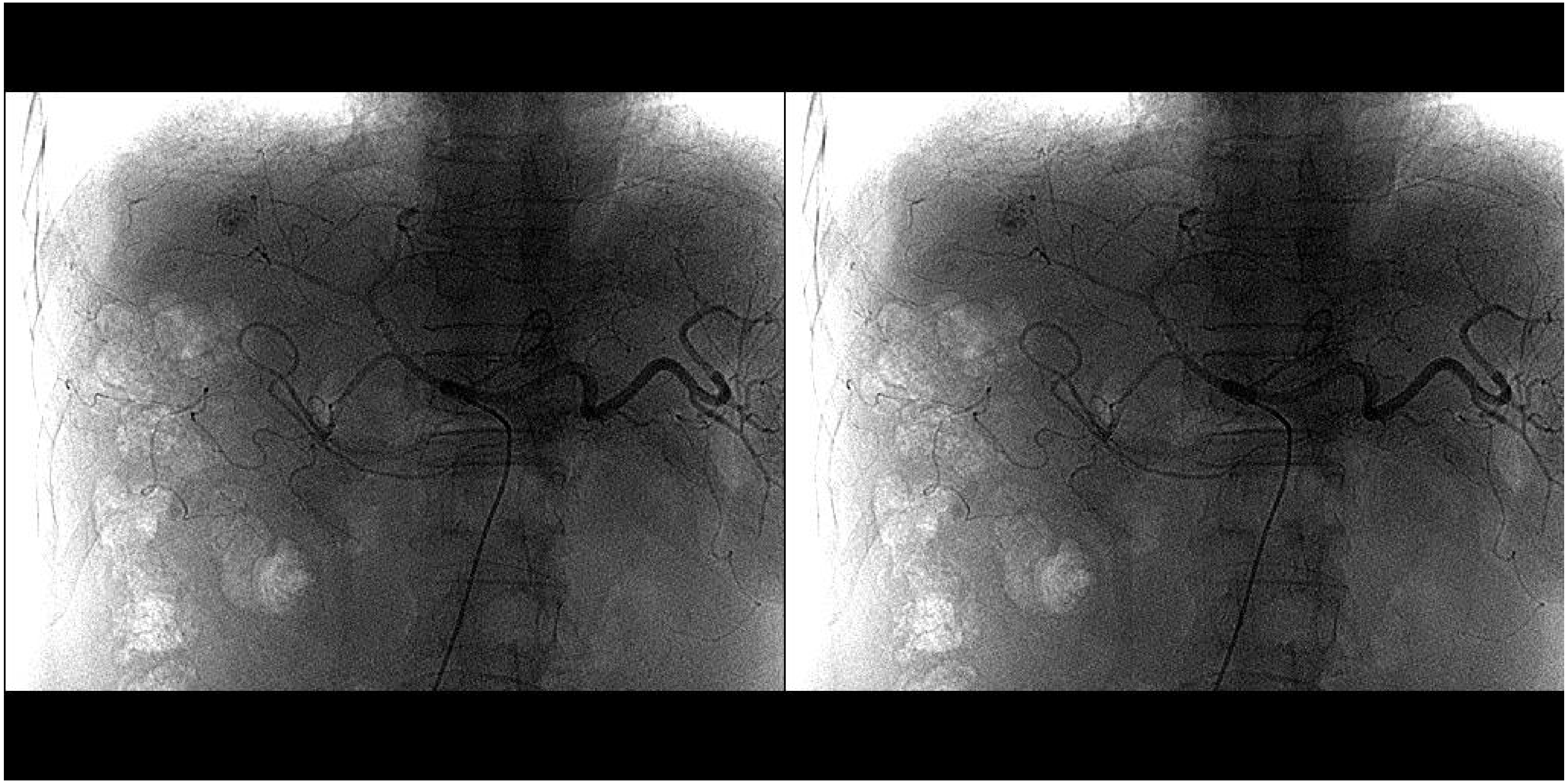

Supplementary Figure S12. Duplicate plain X-ray images from selective angiography of the celiac trunk with different rotation angles (LAO 15.4°/16.1°) were horizontally combined. The α angle between the left and right X-ray images is 0.7°.

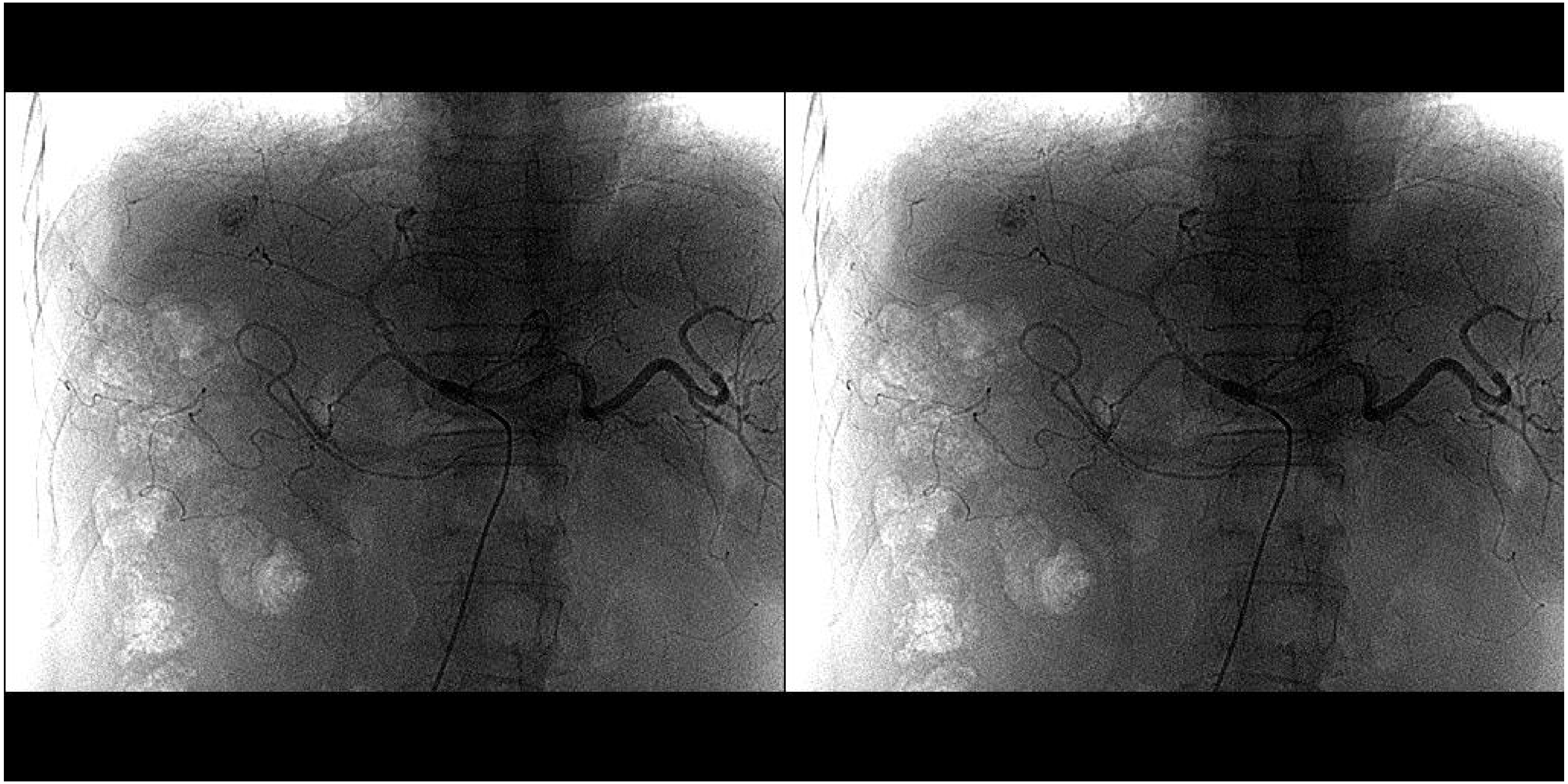

Supplementary Figure S13. Duplicate plain X-ray images from selective angiography of the celiac trunk with different rotation angles (LAO 15.4°/16.8°) were horizontally combined. The α angle between the left and right X-ray images is 1.4°.

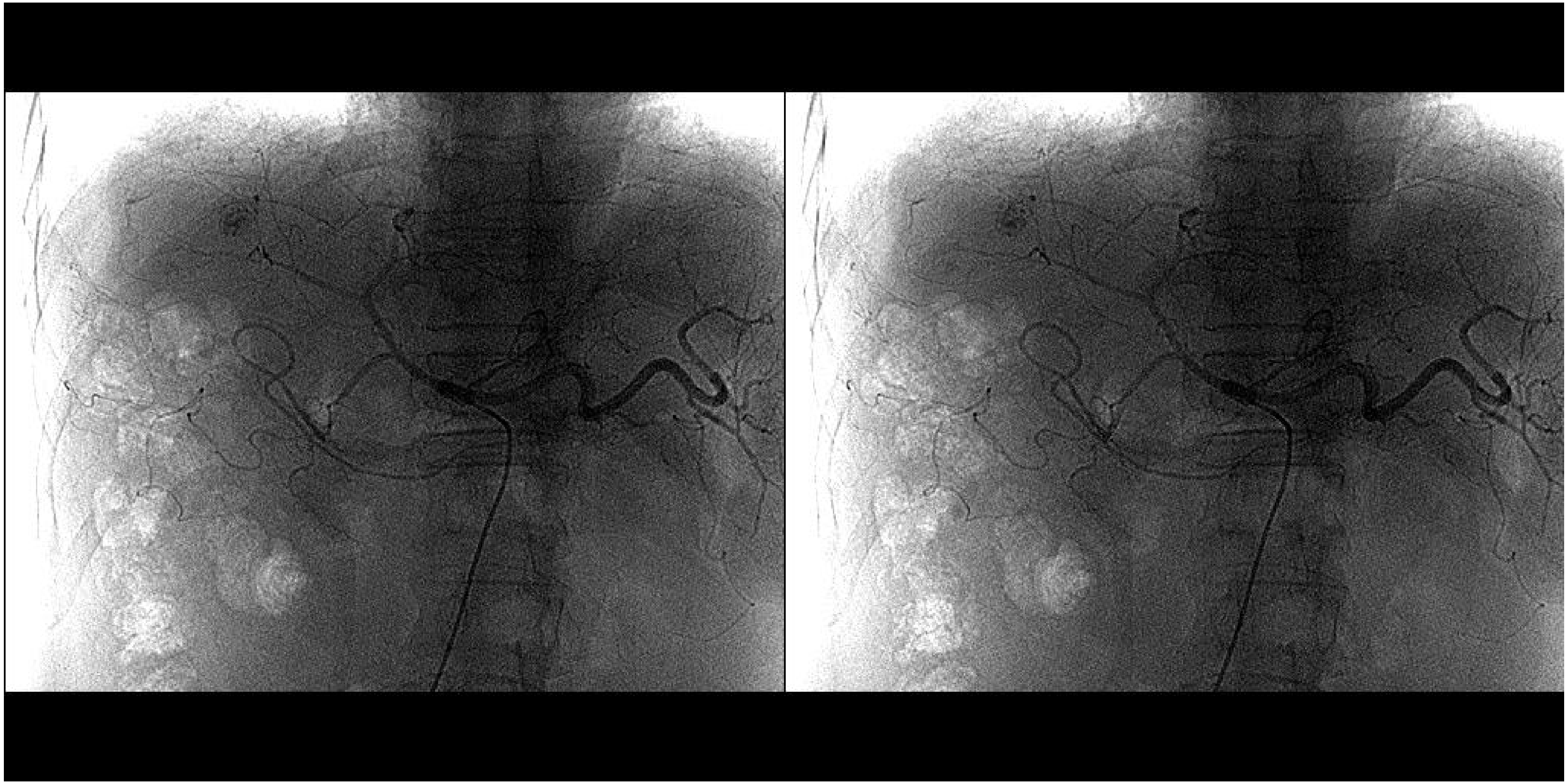

Supplementary Figure S14. Duplicate plain X-ray images from selective angiography of the celiac trunk with different rotation angles (LAO 15.4°/17.5°) were horizontally combined. The α angle between the left and right X-ray images is 2.1°.

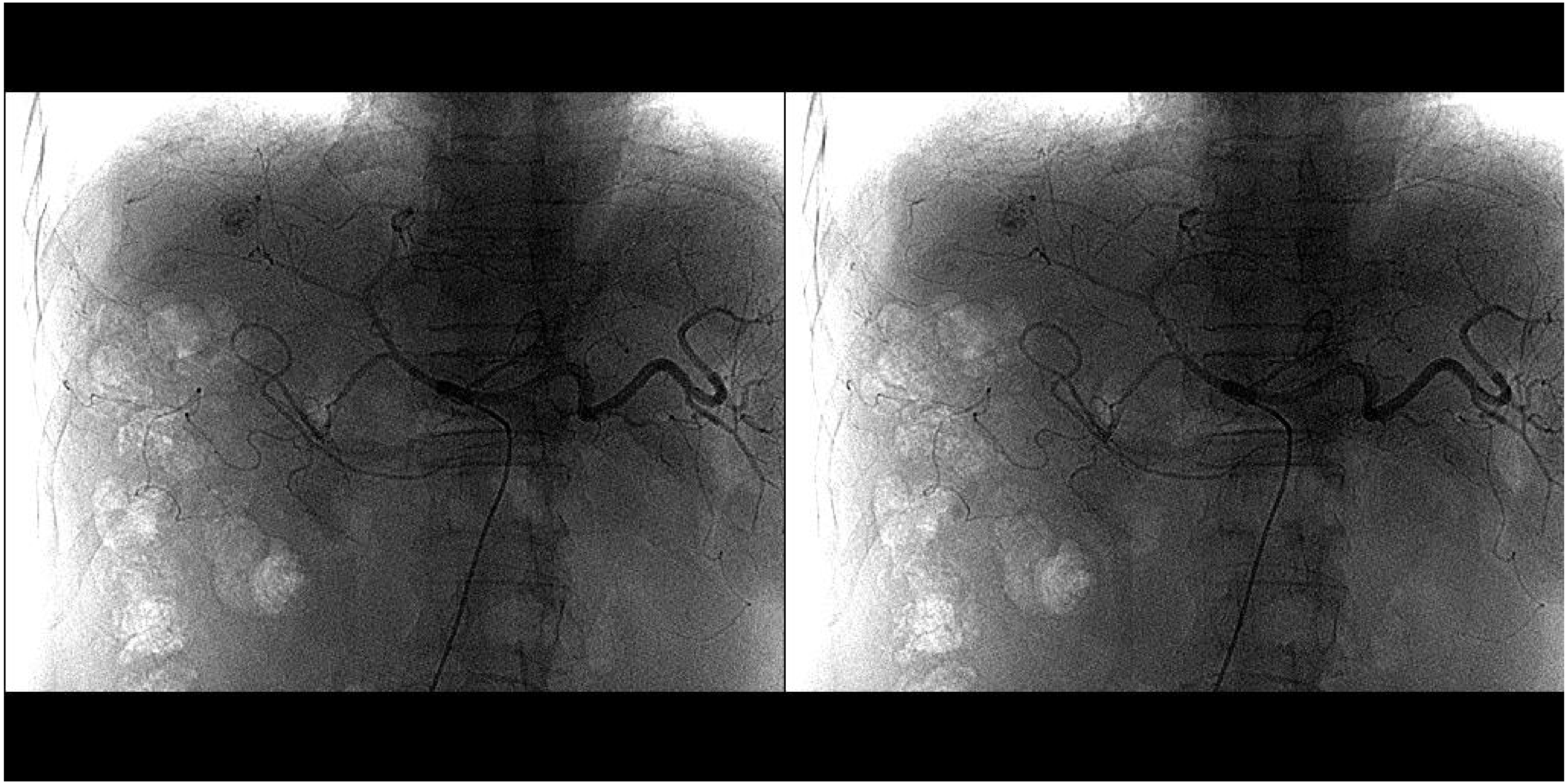

Supplementary Figure S15. Duplicate plain X-ray images from selective angiography of the celiac trunk with different rotation angles (LAO 15.4°/18.2°) were horizontally combined. The α angle between the left and right X-ray images is 2.8°.

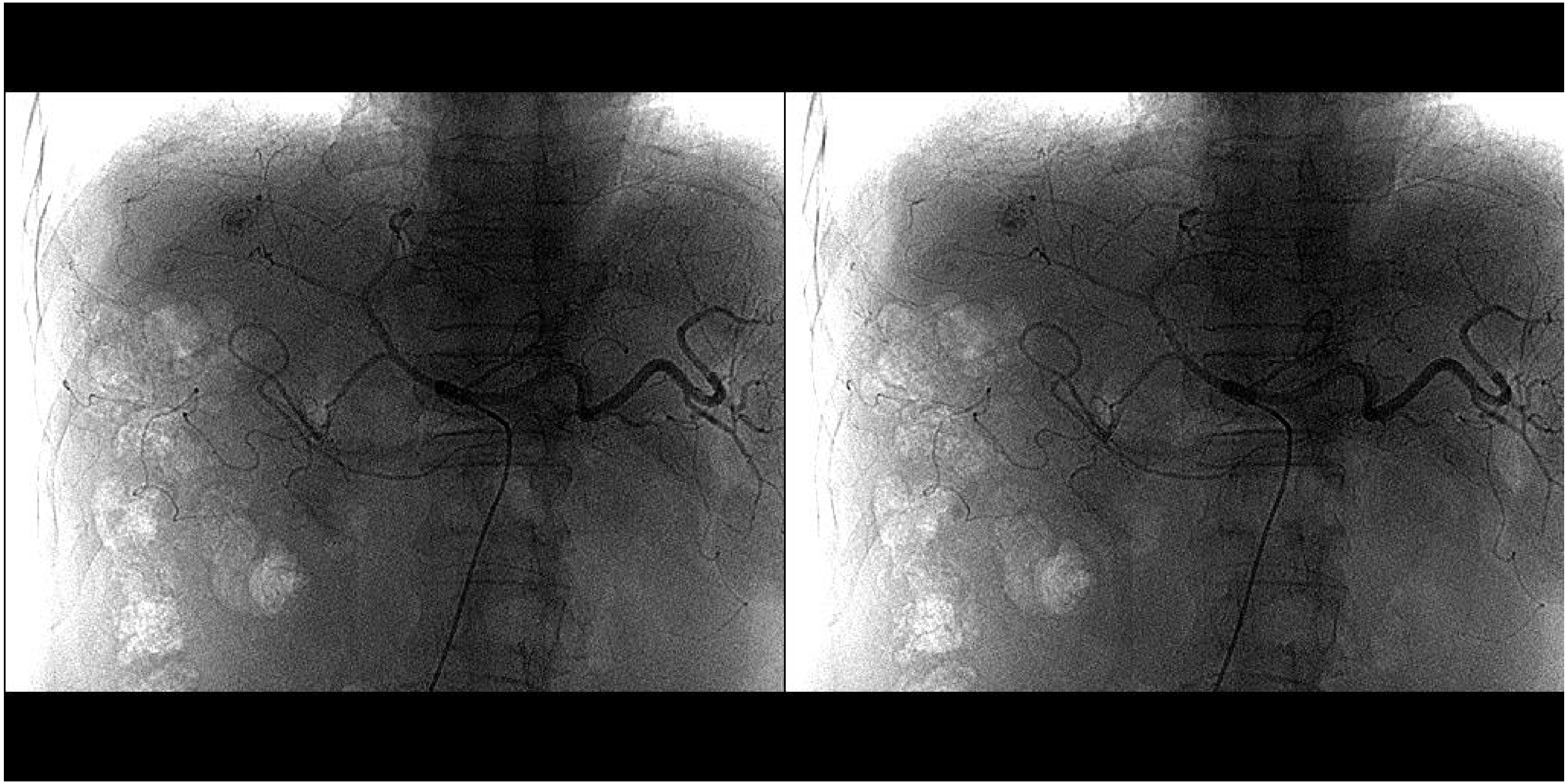

Supplementary Figure S16. Duplicate plain X-ray images from selective angiography of the celiac trunk with different rotation angles (LAO 15.4°/18.8°) were horizontally combined. The α angle between the left and right X-ray images is 3.4°.

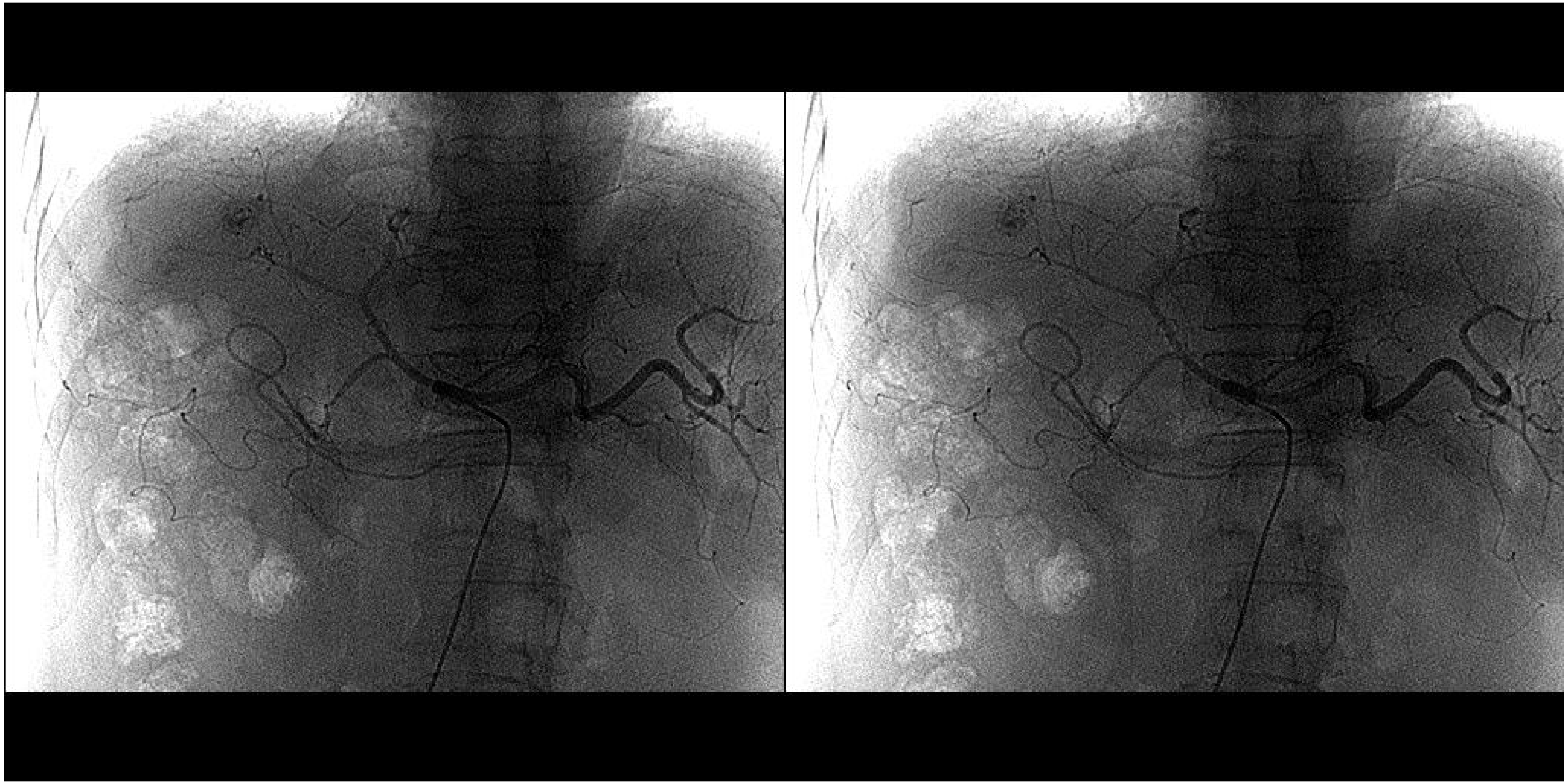

Supplementary Figure S17. Duplicate plain X-ray images from selective angiography of the celiac trunk with different rotation angles (LAO 15.4°/19.5°) were horizontally combined. The α angle between the left and right X-ray images is 4.1°.

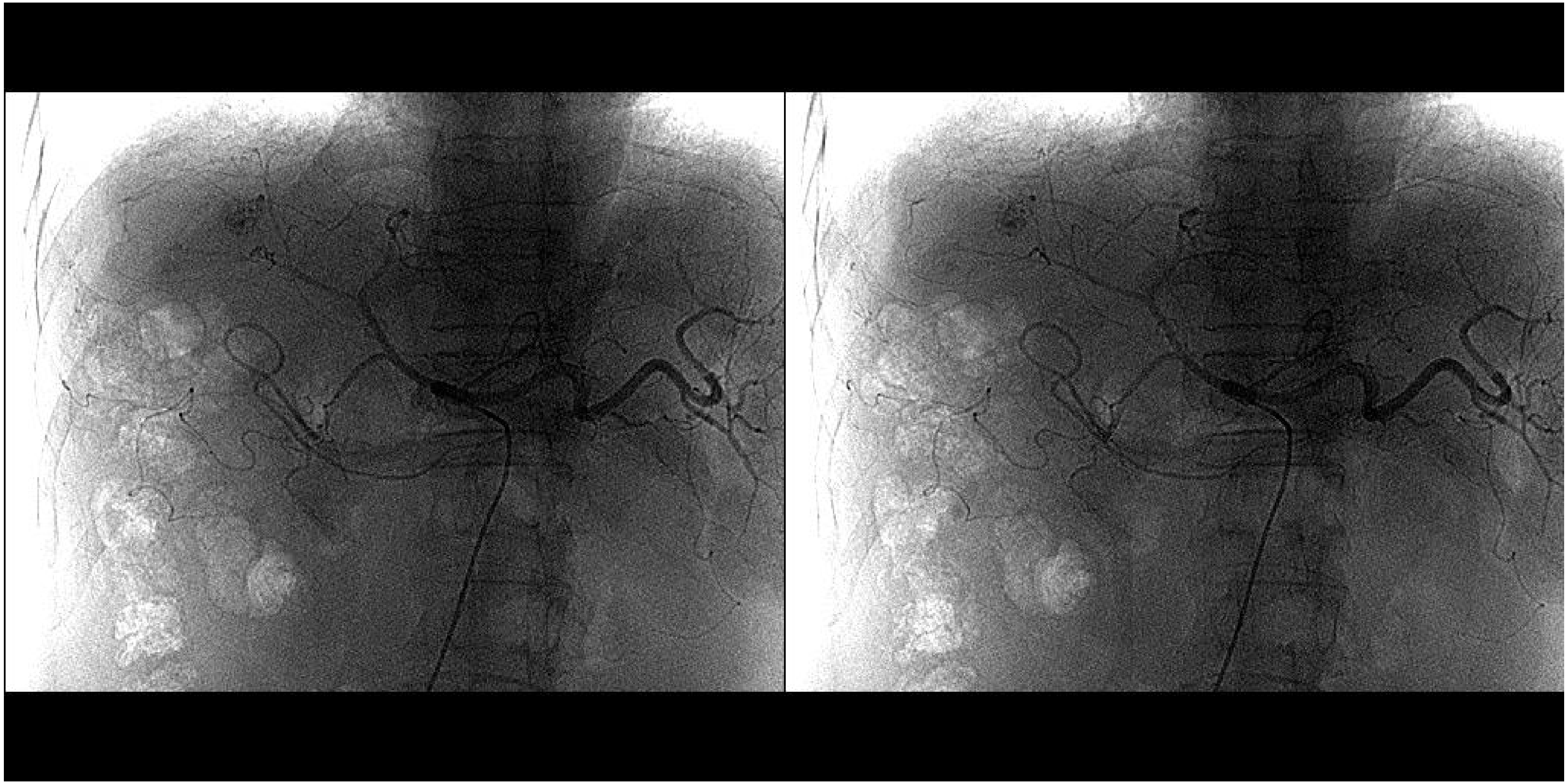

Supplementary Figure S18. Duplicate plain X-ray images from selective angiography of the celiac trunk with different rotation angles (LAO 15.4°/20.2°) were horizontally combined. The α angle between the left and right X-ray images is 4.8°.

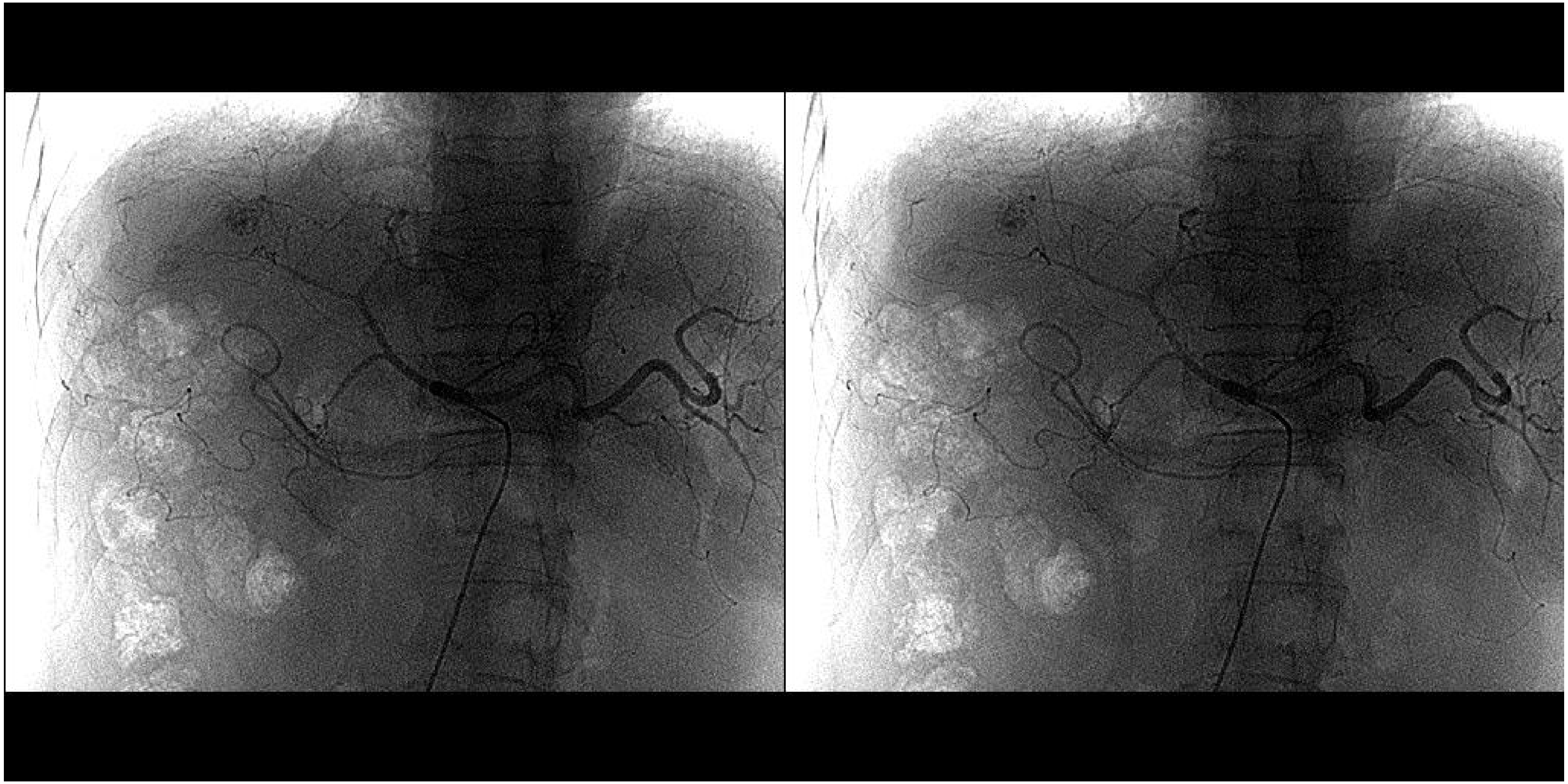

Supplementary Figure S19. Duplicate plain X-ray images from selective angiography of the celiac trunk with different rotation angles (LAO 15.4°/20.9°) were horizontally combined. The α angle between the left and right X-ray images is 5.5°.

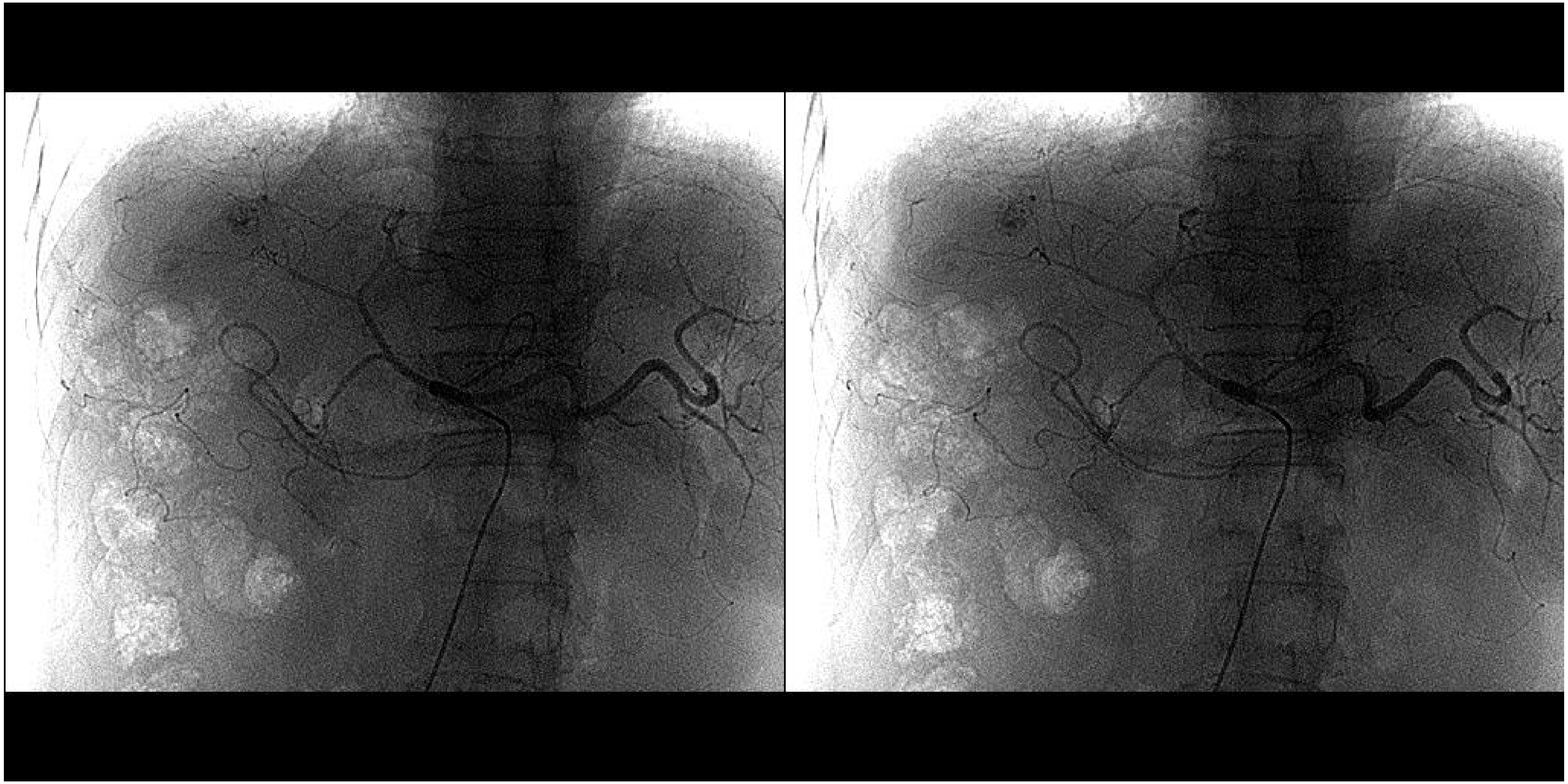

Supplementary Figure S20. Duplicate plain X-ray images from selective angiography of the celiac trunk with different rotation angles (LAO 15.4°/21.6°) were horizontally combined. The α angle between the left and right X-ray images is 6.2°.

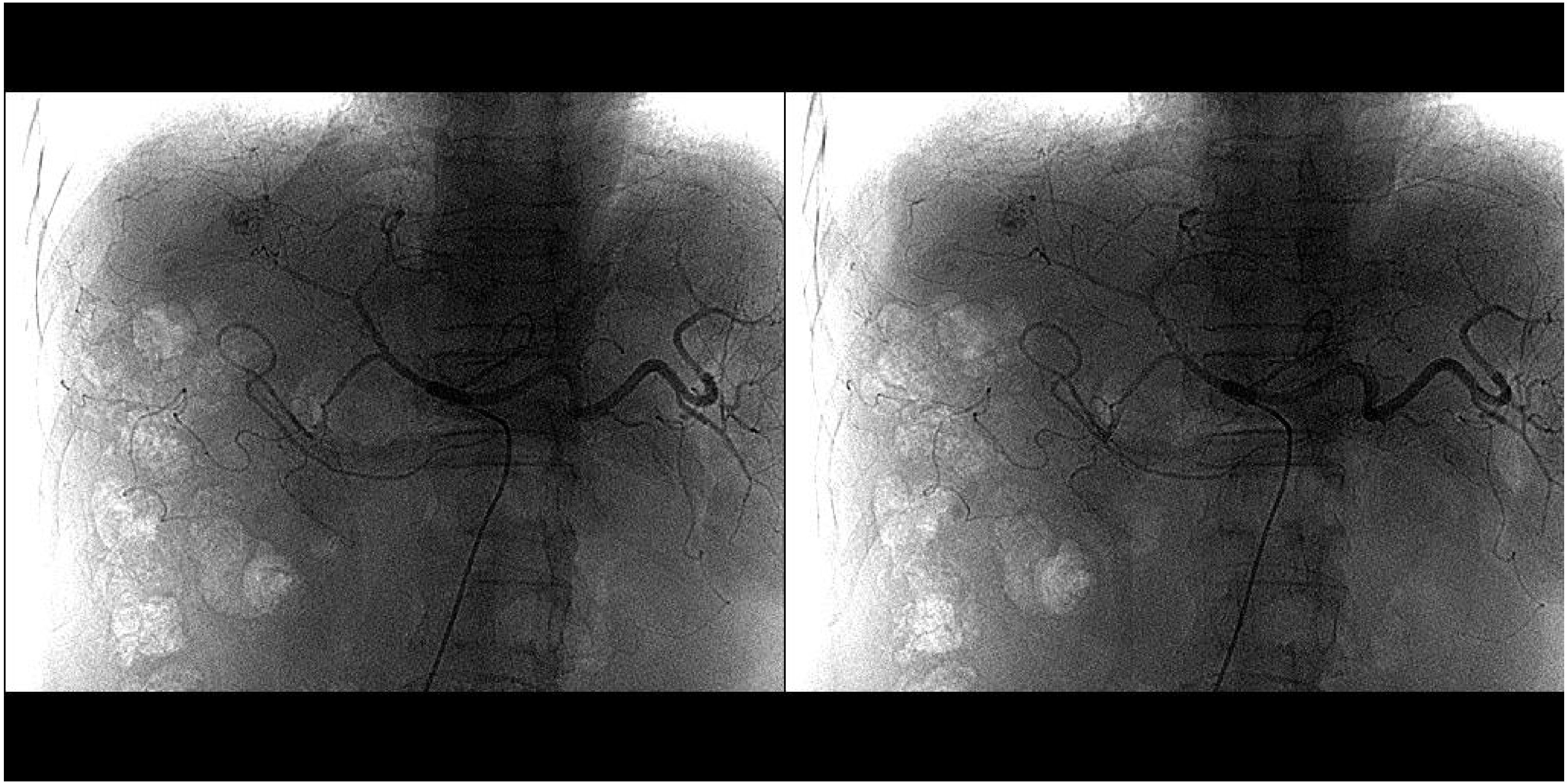

Supplementary Figure S21. Duplicate plain X-ray images from selective angiography of the celiac trunk with different rotation angles (LAO 15.4°/22.2°) were horizontally combined. The α angle between the left and right X-ray images is 6.8°.

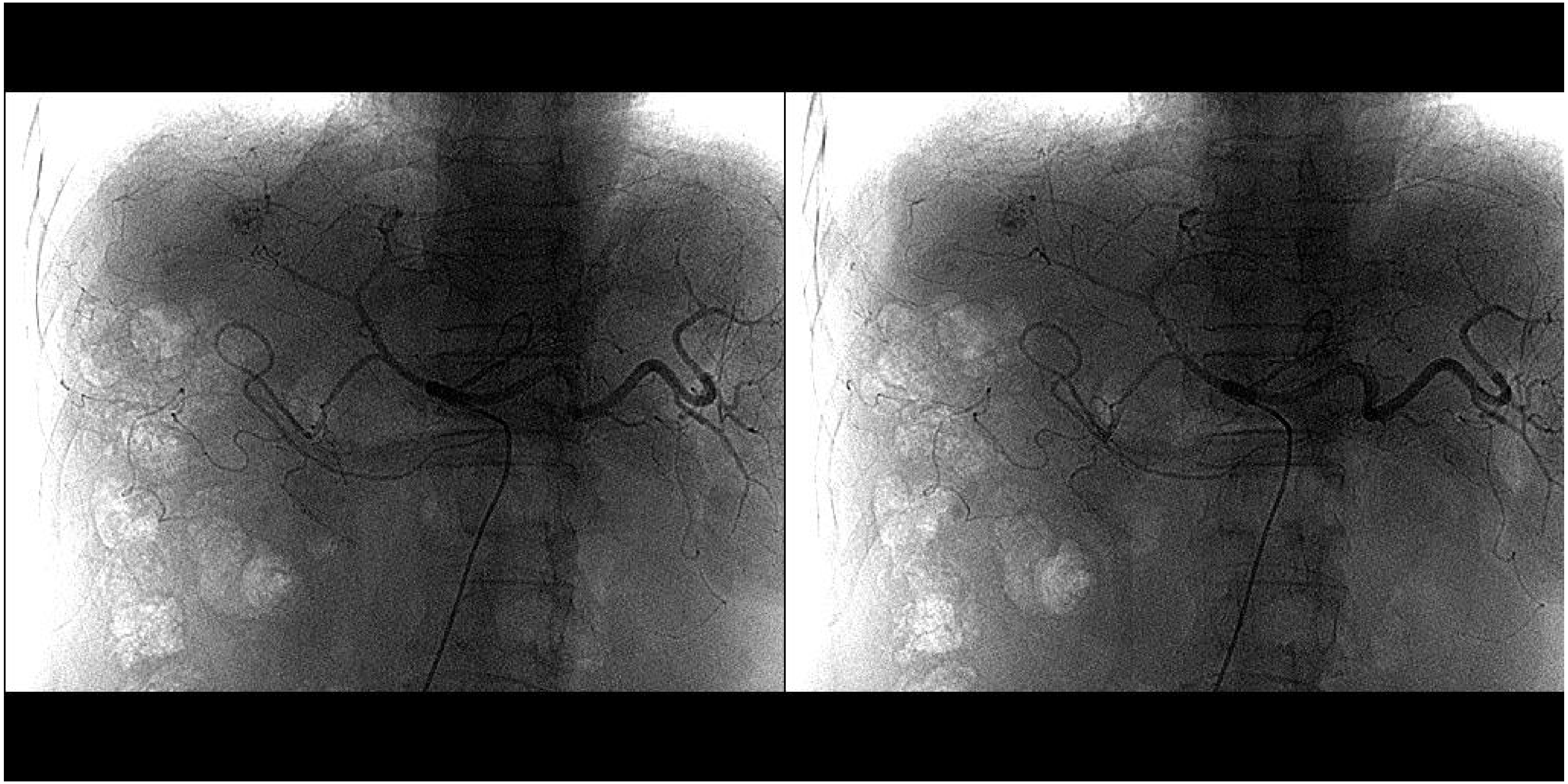

Supplementary Figure S22. Duplicate plain X-ray images from selective angiography of the celiac trunk with different rotation angles (LAO 15.4°/22.9°) were horizontally combined. The α angle between the left and right X-ray images is 7.5°.

Case Three

In another patient with superior mesenteric artery dissection receiving interventional treatment, duplicate plain X-ray videos of superior mesenteric arteriography obtained at the optimal angle (1.8°) during an abdominal roll scan are presented in Supplementary Video S2.

Case Four

The original abdominal computed tomographic angiography (CTA) data from a patient with systemic lupus erythematosus (SLE) were utilized to construct 3D images of the abdomen. Perspective views of the abdomen were prepared according to the law that objects appear larger when nearer to the observer. Duplicate perspective views of the abdomen were horizontally combined in the left-right 3D video (Supplementary Video S4). The α angle between the left and right perspective views is 2°. A more realistic stereo view of abdominal vessels, organs and bones can be obtained with a pair of VR glasses.

Case Five

The original magnetic resonance cholangio-pancreatography (MRCP) data from a patient with obstructive jaundice were utilized to construct 3D images of the pancreaticobiliary system. Perspective views of the pancreaticobiliary system were prepared according to the law that objects appear larger when they are nearer to the observer. Duplicate perspective views of the pancreaticobiliary system were horizontally combined in the left-right 3D video (Supplementary Video S5). The α angle between the left and right perspective views is 2°. A more realistic stereo view of the pancreaticobiliary system can be obtained with a pair of VR glasses.

Supplementary Videos

Supplementary Video S1. Duplicate plain X-ray videos of external carotid angiography with an α angle of 1.8° during a cerebral prop scan.

Supplementary Video S2. Duplicate plain X-ray videos of superior mesenteric arteriography with an α angle of 1.8° during an abdominal roll scan.

Supplementary Video S3. Duplicate plain X-ray videos with an α angle of 2.4° during direct portography.

Supplementary Video S4. Duplicate perspective views of the abdomen were horizontally combined in the left-right 3D video. The α angle between the left and right perspective views is 2°. A more realistic stereo view of abdominal vessels, organs and bones can be obtained with a pair of VR glasses.

Supplementary Video S5. Duplicate perspective views of the pancreaticobiliary system were horizontally combined in the left-right 3D video. The α angle between the left and right perspective views is 2°. A more realistic stereo view of the pancreaticobiliary system can be obtained with a pair of VR glasses.

In Supplementary Videos 4 and 5, the tissues and vessels in the left-eye and right-eye X-ray images appeared larger when nearer and smaller when farther away, according to the distance ratio. The α angle between the left and right X-ray images is 2°. Observation with VR glasses can produce more realistic stereo views of structures such as vessels, bones, and tissues. The results demonstrate that the simple law of perspective that objects appear larger when nearer to the observer is helpful for generating more realistic stereo X-ray images. Introduction of the simple law of perspective into the X-ray images is necessary for generating realistic stereo vision. Using a point X-ray source will achieve this goal by ensuring that the tissue shadow in the perspective view is larger when the tissue is nearer and smaller when it is farther away, according to the distance ratio. Therefore, a point X-ray source is useful for improving the stereo fidelity of the technique.
